# Polysorbates versus Hydroxypropyl Beta-Cyclodextrin (HPβCD): Comparative Study on Excipient Stability and Stabilization Benefits on Monoclonal Antibodies

**DOI:** 10.3390/molecules27196497

**Published:** 2022-10-01

**Authors:** Hailong Zhang, Shiqi Hong, Sarah Si Kai Tan, Tao Peng, Lucas Yuan Hao Goh, Kwan Hang Lam, Keat Theng Chow, Rajeev Gokhale

**Affiliations:** 1 Pharma Applied Sciences, Roquette Asia Pacific Pte Ltd., Singapore 138588, Singapore; 2 Global Pharmaceutical Sciences, Roquette America Inc., 2211 Innovation Drive, Geneva, IL 60134, USA

**Keywords:** polysorbate, 2-hydroxylpropyl-β-cyclodextrin, KLEPTOSE^®^ HPB, monoclonal antibody, protein stability, formulation design, stress study, adalimumab

## Abstract

Polysorbates (PS 20 and PS 80) are the most widely used surfactants in biopharmaceutical formulations to protect proteins from denaturation, aggregation, and surface adsorption. To date, around 70% of marketed therapeutic antibodies contain either PS 20 or PS 80 in their formulations. However, polysorbates are chemically diverse mixtures, which are prone to degradation by oxidation and hydrolysis to produce peroxides and fatty acids, which, in turn, induce protein oxidation, aggregation, and insoluble particle formation. These will negatively impact protein quality and stability. Thus, polysorbate degradation has emerged as one of the major challenges in the development and commercialization of therapeutic protein products. KLEPTOSE^®^ HPβCD (hydroxypropyl beta-cyclodextrin), a new multifunctional excipient, has been shown to provide protein stabilization functions in biopharmaceutical downstream processes and in their final formulations. This study aims to evaluate HPβCD, a new molecule of its class, against polysorbates as a stabilizer in biologics formulations. In this study, the chemical stability of KLEPTOSE^®^ HPβCDs is compared with polysorbates (20 and 80) under various stress conditions. When subjected to heat stress, HPβCDs show little change in product recovery (90.7–100.7% recovery for different HPβCDs), while polysorbates 20 and 80 show significant degradation, with only 11.5% and 7.3% undegraded product remaining, respectively. When subjected to other chemical stressors, namely, autoclave, light, and oxidative stresses, HPβCD remains almost stable, while polysorbates show more severe degradation, with 95.5% to 98.8% remaining for polysorbate 20 and 85.5% to 97.4% remaining for polysorbate 80. Further, profiling characterization and degradation analysis reveal that chemical structures of HPβCDs remain intact, while polysorbates undergo significant hydrolytic degradation and oxidation. Lastly, the physicochemical stability of monoclonal antibodies in formulations is investigated. When subjected to light stress, adalimumab, as a model mAb, formulated in the presence of HPβCD, shows a significant decrease in protein aggregation, and superior monomer and total protein recovery compared to PS 80-containing formulations. HPβCD also reduces both agitation and thermal stress-induced protein aggregation and prevents subvisible particle formation compared to PS 80.

## 1. Introduction

Antibody-based proteins such as monoclonal antibodies (mAbs) have become a dominant group of therapeutics in the fast-growing biopharmaceuticals sector. A major challenge during formulation development of these biotherapeutic proteins is overcoming their limited stability. A robust and stable formulation is thus essential to ensure stability, efficacy, and safety of these biotherapeutic proteins. Among the various protein degradation pathways, the formation of protein aggregates and particles is of particular concern [1]. 

Surfactants are commonly added to protein formulations to mitigate interfacial-induced aggregation and surface adsorption experienced during commercial processing steps, transportation, and clinical administration [2,3]. Surfactants are thought to protect proteins against interfacial damage by competing with proteins for adsorption sites on different interfaces, such as air–water, solid–water, and oil–water interfaces [4,5]. Owing to their decades of parenteral use in humans and demonstrated safety [6], polysorbate 80 (PS 80) and polysorbate 20 (PS 20) are considered the choice surfactants used in more than 90% of approved mAbs products [7]. However, in recent years, there have been increasing concerns about polysorbate degradation and its impact on the safety and quality of these biotherapeutic products. PS 20 and PS 80 are composed of heterogeneous mixtures of structurally related fatty-acid esters of polyoxyethylene (POE) sorbitan and lauric acid or oleic acid, respectively [8]. Polysorbates are prone to degradation by auto-oxidation and hydrolysis [9]. The ester bond in polysorbates can be hydrolyzed when promoted by heat and catalyzed by acid, base, or enzymes such as esterases and lipases. Hydrolysis, especially enzymatically driven, leads to the formation of free fatty acids as degradation products. Polysorbate can also be oxidized through exposure to light or by residual peroxides from manufacturing, or by transition metals, leading to a diversity of degradation products such as peroxides, aldehydes, and alkanes. These degradation products can lead to protein aggregation and formation of visible and subvisible particles. Particle formation has recently emerged as a major concern in formulations. The regulatory health authorities require biopharmaceutical companies to closely monitor the visible and subvisible particle formation by comprehensive characterization, as outlined in the United States Pharmacopeia (USP)−USP 〈787〉, USP 〈788〉, and USP 〈789〉. The accumulation of free fatty acids and subsequent formation of subvisible and visible particulates in the polysorbate formulations have been reported in numerous studies [10,11,12,13]. The presence of protein aggregates and particles not only compromise product quality and long-term stability, but they may also cause unwanted immune responses [14]. Consequently, this has led to an increased interest in searching for more stable surfactant alternatives. 

**Figure 1:**
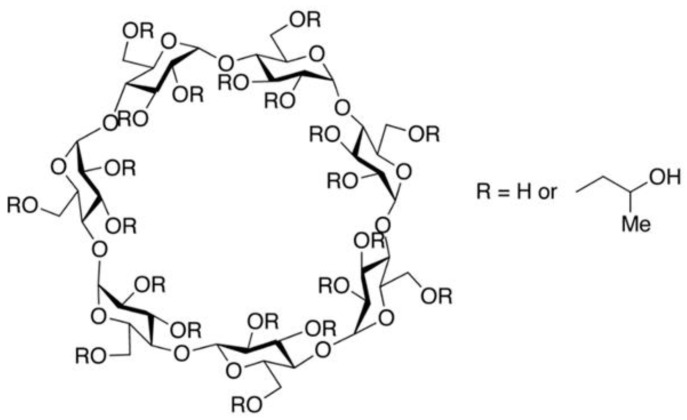
Chemical structure of hydroxypropyl-beta-cyclodextrin.

Among the potential candidates, hydroxypropyl β-cyclodextrin (HPβCD) has emerged as an interesting class of molecule. HPβCD is known to possess surface activity [15,16] and has been explored as a functional alternative to polysorbates in biologics formulations [17,18]. HPβCD is one of the derivatives of β-CD. Native β-cyclodextrin and its derivatives are cyclic oligosaccharides obtained from starch by enzymatic cyclization [19]. They are composed of seven α-glucopyranose monomers, forming a unique torus-like shaped structure, with a hydrophilic outer surface and a hydrophobic internal cavity. HPβCD is a substituted hydroxypropyl ether of β-cyclodextrin in alkaline conditions (Figure 1). β-cyclodextrin has a total of 21 hydroxyl groups, which gives rise to a huge number of possible combinations of substitutions per molecule. Roquette has developed a range of substituted hydroxypropyl-β-cyclodextrins with different degrees of substitution that are described by the molar substitution level. The key points of each grade of KLEPTOSE® HPβCD used in this study are listed in Table 1. Substitution of the hydroxyl groups in β-CD with hydroxypropyl groups results in high water solubility of HPβCD. Depending on the degree of substitution (DS), i.e., number of substituted hydroxyl groups in a glucose moiety of the CD molecule, the exact physicochemical properties of HPβCD can differ slightly [20]. The resulting HPβCD is a well-established excipient used in many approved small molecule drug products and is included in the United States Pharmacopeia (USP) and European Pharmacopoeia (Pharm. Eur.). Due to its regulatory status as an approved excipient for oral and parenteral administration [21], HPβCD has also been widely explored as a stabilizer in biologic formulations [22,23]. Several studies have shown HPβCD to effectively prevent protein aggregation in human growth hormone [24], human plasma immunoglobulin G (IgG) [25], and several mAbs by preventing either thermal or agitation-induced aggregation [26] in solution as well as lyophilization-related aggregation [27,28]. Recent studies have also demonstrated HPβCD to be effective in reducing interfacial stress-induced particle formation during ultrafiltration/diafiltration [29,30,31]. A few mechanistic studies have been conducted to understand the stabilization mechanism of HPβCD in the biopharmaceutical formulations. Among these studies, it was postulated that the stabilizing effect of HPβCD is attributed to its ability to reduce protein–protein interactions by shielding hydrophobic interactions [26,32]. Though not as efficient as classical surfactants [18], HPβCD may also displace protein from interfaces due to its weak surface activity. These unique stabilization properties make HPβCD a potential functional excipient in biologics formulations.

As described, physicochemical stability issues related to PS 80 and PS 20 are major concerns. Therefore, to qualify HPβCD as a potential alternative, the stability of HPβCD in formulations needs to be assessed. In this work, we evaluated for the first time the chemical stability of HPβCD in aqueous medium under various pharmaceutically relevant stressed conditions such as thermal, light, autoclave, and oxidation stresses. A direct stability comparison was performed by subjecting three grades of HPβCD, PS 80, and PS 20 to the same stress conditions. An array of analytical techniques such as refractive index detection (RID), charged aerosol detection (CAD), and mass spectrometry (MS) was employed to quantify and characterize the parent chemicals and their degradants. 

In addition to physicochemical stability, we also compared the efficiency of HPβCD and PS 80 in preventing protein aggregation and particle formation under various stress conditions. Adalimumab was chosen as a model protein for this work, as its commercial formulation contains PS 80, and it is reported to be susceptible to light and shear stresses. The different adalimumab formulations containing either HPβCD or PS 80 or a combination of both were subjected to stirring and thermal and light stresses. Particle (subvisible and nano) aggregation and fragmentation as well as charge heterogeneity in different formulations were then quantitatively characterized using micro-flow imaging (MFI), dynamic light scattering (DLS), size exclusion chromatography (SEC), and cation-exchange chromatography (CEX). 

Overall, this work provides a comprehensive comparison of HPβCD and polysorbates with respect to their chemical stability and their efficiency in protecting biologics from stress-induced aggregation and particle formation. Quantitative analysis and molecular profiling characterization have shown that KLEPTOSE® HPβCD products exhibited greater stability than polysorbates and effectively protected monoclonal antibodies and prevented subvisible particle formation. Our study of the stability comparison between HPβCD and polysorbates and the stabilization benefits of monoclonal antibodies indicates that HPβCD may be considered as an effective alternative to polysorbates for protein stabilization.

## 2. Results

### 2.1. Accelerated Thermal Stability Studies

**Figure 2:**
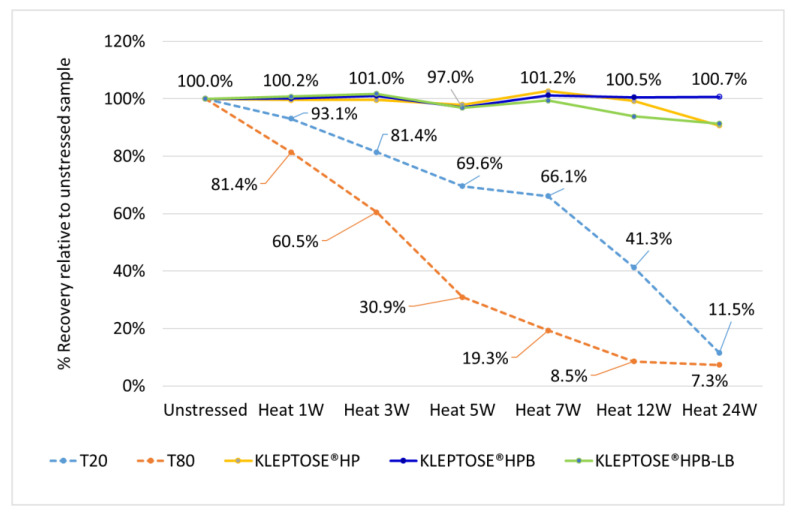
Thermal stability study of polysorbates and KLEPTOSE® HPβCD at 40 °C/75% RH within 24 weeks (W).

To compare stability of polysorbates and HPβCD under thermal stress, a six-month accelerated stability testing was carried out at 40 °C/75% RH per ICH guidelines. Two polysorbates (PS 20 and PS 80) and three HPβCDs (KLEPTOSE^®^ HP, HPB, and HPB-LB) were formulated and tested for stability evaluation. When subjected to mild thermal stress at 40 °C until 24 weeks, different grades of KLEPTOSE^®^ HPβCD showed slight changes in product recovery (90.7%, 100.7%, and 91.4% for KLEPTOSE^®^ HP, HPB, and HPB-LB, respectively), while PS 20 and PS 80 suffered significant degradation with the product recovery of 11.5% and 7.3%, respectively (Figure 2).

**Figure 3:**
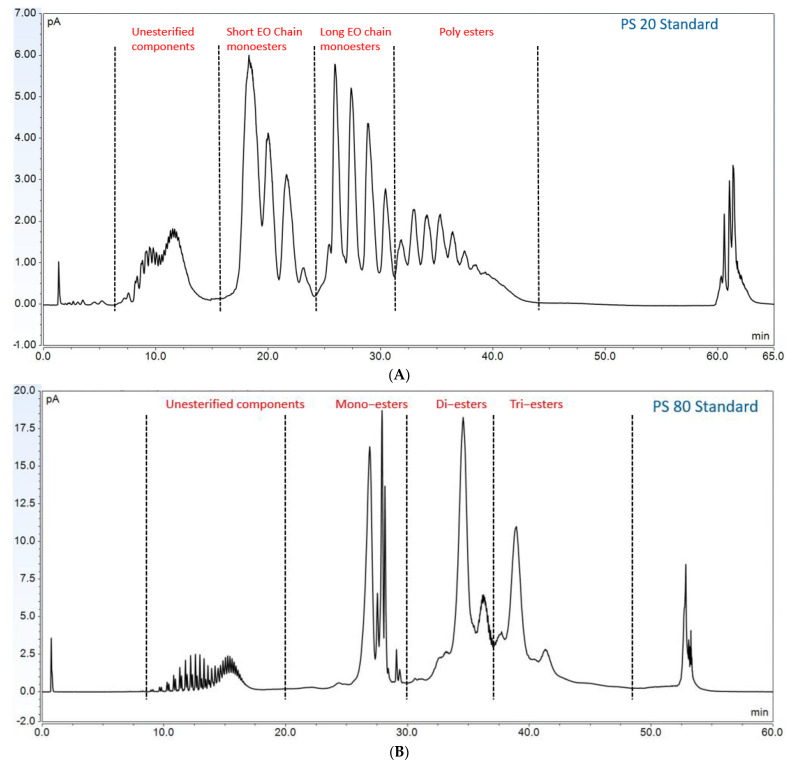

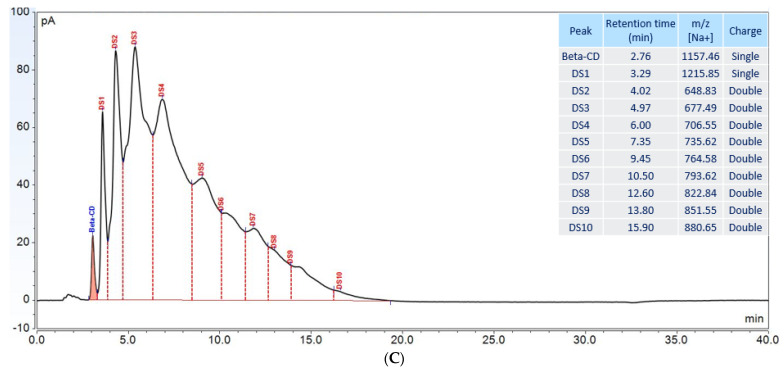
CAD chromatography of PS 20 (**A**), PS 80 (**B**), and KLEPTOSE^®^ HPB (**C**).

Furthermore, to gain insight into the degradation pathways and degradation products, an HPLC-based separation method with a combination of charge aerosol detector (CAD) and mass spectrometer (MS) was developed to examine the chemical components of polysorbates and KLEPTOSE^®^ HPβCD and their degradants. The component profiles obtained by CAD for PS 20, PS 80, and KLEPTOSE^®^ HPβCD are shown in Figure 3, where a single quadrupole mass detector was used to elucidate the nature of the species eluting in the different groups (Table 2). Four different groups of peak clusters from PS 20 were identified as non-esterified components, short ethylene oxide (EO) chain monoesters, long EO chain monoesters, and polyesters (di- and tri-esters), while non-esterified components, monoesters, diesters, and tri-esters were major groups in PS 80. Generally, the KLEPTOSE^®^ HPβCD was characterized by the degree of substitution (DS)—the average number of substituents on a cyclodextrin (CD) molecule. With the help of the mass spectrometer, a complex mixture of HPβCD species with differing degrees of hydroxy-propylation (HP) were elucidated, in which parental β-cyclodextrin and HP-substituted β-cyclodextrin derivatives (10 substituents in KLEPTOSE^®^ HPB and KLEPTOSE^®^ HPB-LB, 12 in KLEPTOSE^®^ HP) were identified. 

**Figure 4:**
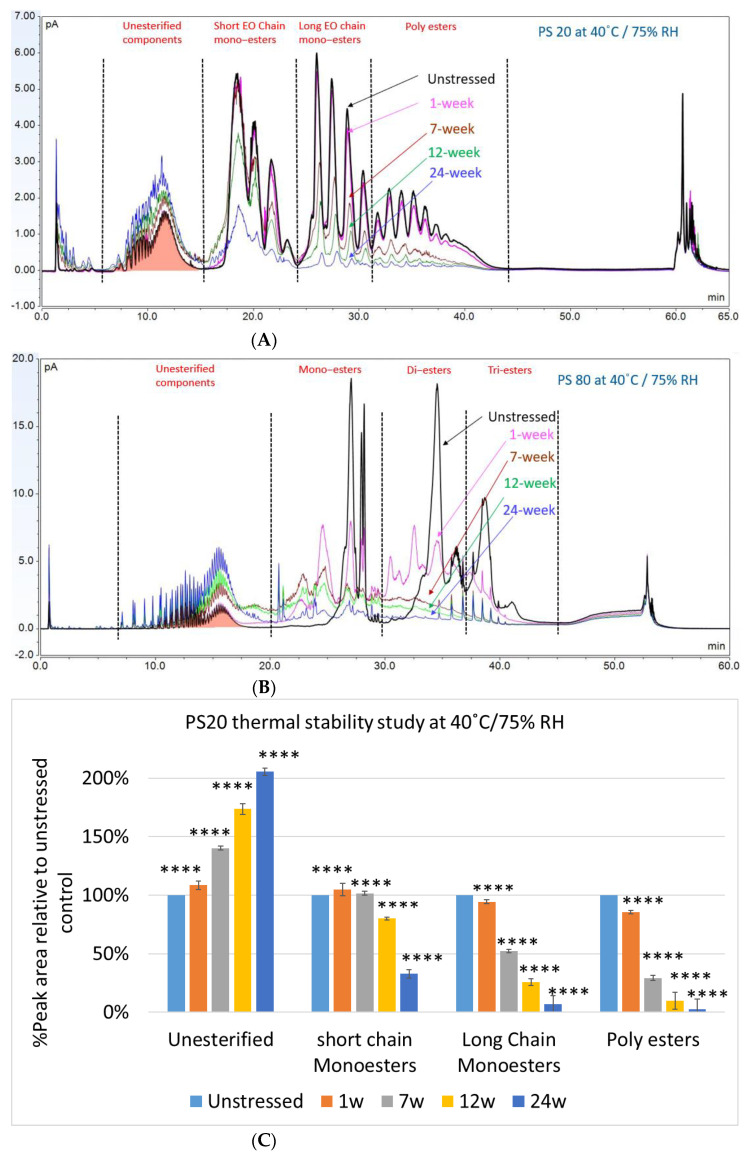

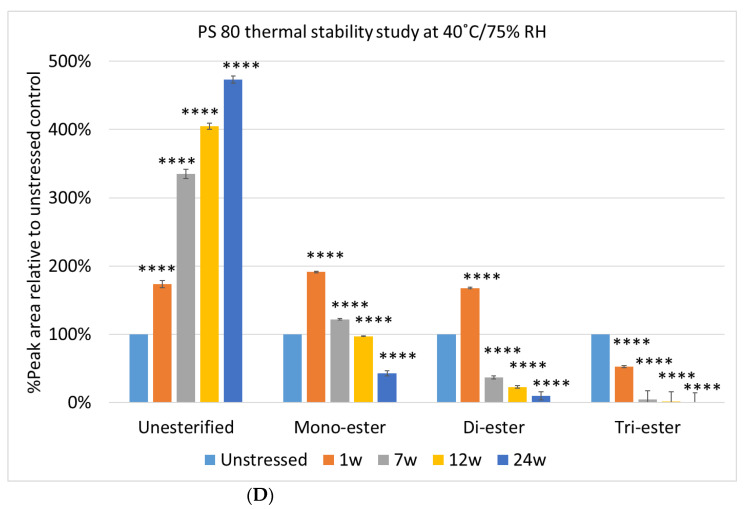
Thermal stability study of PS 20 and PS 80 at 40 °C / 75% RH: overlay chromatogram of PS 20 (**A**) and PS 80 (**B**) over time; the degradation of POE sorbitan esters in PS 20 (**C**) and in PS 80 (**D**) over time. **** *p* < 0.0001.

In this accelerated stability study, peak areas of each group for PS 20, PS 80, and HPβCD at different time points were analyzed to compare stability differences among them. As shown in Figure 4, analysis of chromatograms showed very rapid degradation of PS 20 under thermal stress; the main components of esters, including monoesters and polyesters, decreased over time (short EO chain ester down to 33%, long EO chain down to 7%, and polyesters down to 3%, respectively), while unesterified components (POE, POE isosorbide, and POE sorbitan) increased up to 206% compared to the unstressed sample. Similarly, PS 80 also exhibited significant degradation within a 24-week period under thermal stress, in which main components, namely, mono-, di-, and tri-ester, released more fatty acids (43% for monoesters, 10% for diesters, and 1% for tri-esters) and accumulated the unesterified components POE, POE isosorbide, and POE sorbitan over time (up to 473%). Interestingly, monoesters in PS 20 remained stable during the first 7 weeks of thermal stress but started to decrease after that. On the other hand, levels of mono−esters and di-esters in PS 80 increased within one week of heat stress before decreasing. This temporary buildup of mono-and di-esters in both PS 20 and PS 80 resulted from the degradation of polyesters, which suggested that both PS 20 and PS 80 degrade through hydrolysis under heat stress [13]. This observation is consistent with the change of overall concentration of PS 20 and PS 80 determined previously. The correlation coefficients for the degradation of PS 20 and PS 80 with accelerated thermal stress was also examined. Statistical analysis indicated that thermal stress at 40 °C / 75% RH led to significant degradation of major components (POE sorbitan esters) in both PS 20 and PS 80. 

**Figure 5:**
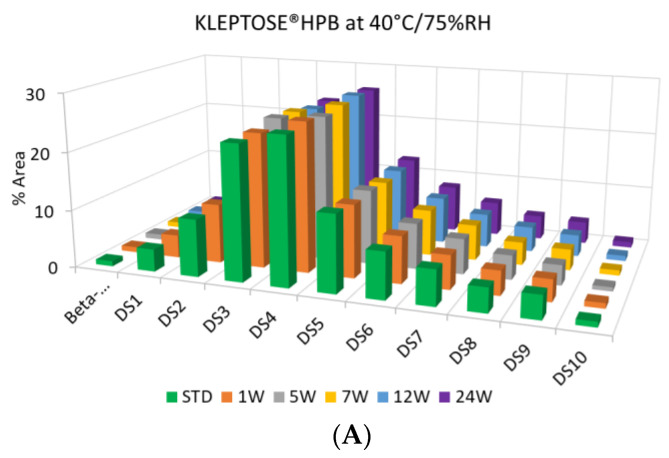

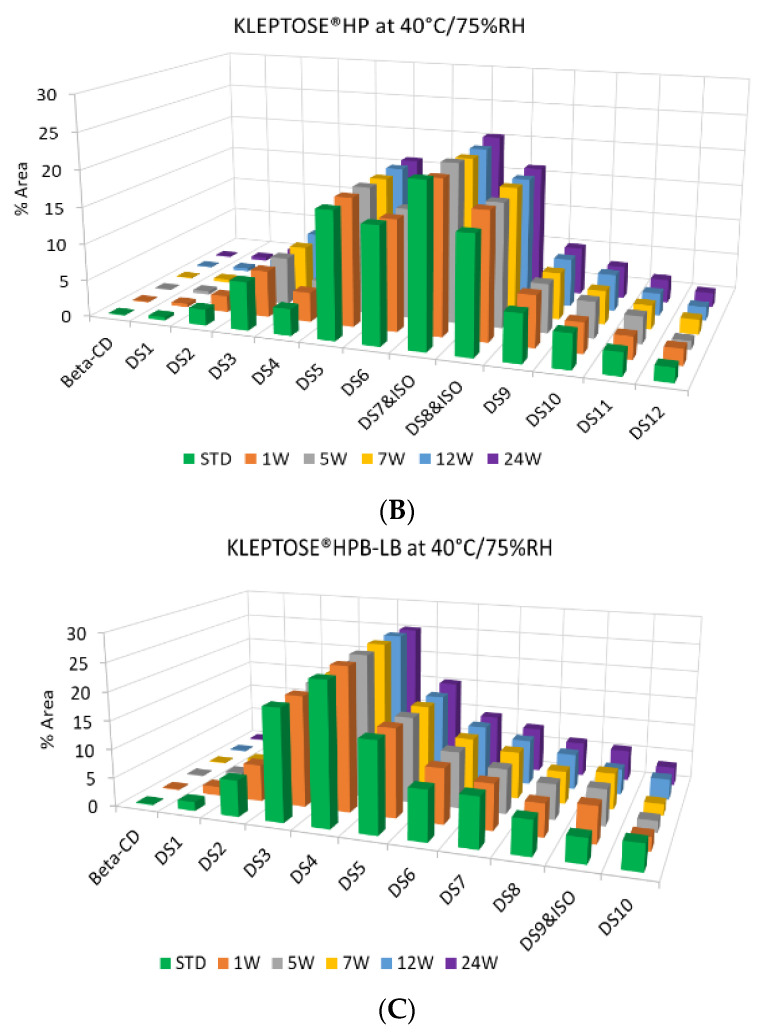
Molecular profiling of representative KLEPTOSE^®^ HPB (**A**), KLEPTOSE^®^ HP (**B**), and KLEPTOSE^®^ HPB-LB (**C**) under mild thermal stress (40 °C / 75% RH) for up to 24 weeks.

To evaluate the degradation level of KLEPTOSE^®^ HPβCD under thermal stress, the change in each substituent was analyzed by measuring their amount (calculated by peak area) at different time points. As shown in Figure 5A, KLEPTOSE^®^ HPB exhibited great stability without obvious change for each cyclodextrin substituent within 24 weeks. Similarly, KLEPTOSE^®^ HP (Figure 5B) and HPB-LB (Figure 5C) showed comparable stability under thermal stress to that of KLEPTOSE^®^ HPB. The monitoring of each hydroxypropyl substituent provides full insight and a more complete understanding of the stability of KLEPTOSE^®^ HPβCD, especially considering that HPβCD is a complex mixture of distinct species from variations in production.

### 2.2. Forced Degradation Studies

**Figure 6:**
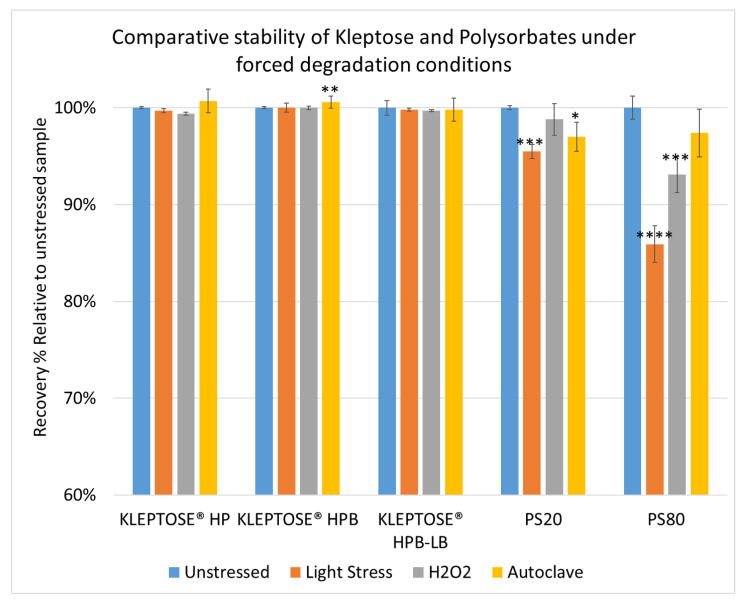
Comparative stability of KLEPTOSE^®^ HP, HPB and HPB-LB, PS 20, and PS 80 under autoclave, oxidative, and light stresses. * *p* < 0.05, ** *p* < 0.01, *** *p* < 0.001, and **** *p* < 0.0001.

To further understand the chemical stability of polysorbates and KLEPTOSE^®^ HPβCD under more severe conditions than accelerated thermal stress conditions, forced degradation studies were carried out to determine the intrinsic stability of both polysorbates and KLEPTOSE^®^ HPβCD. The stress conditions chosen for forced degradation studies included thermal degradation (autoclave, 121 °C), photolysis (illumination of 1.2 million lux hours and an integrated near ultraviolet energy of 200-watt hours/square meter), and oxidation (0.3% H_2_O_2_). We first evaluated the overall sample concentration of polysorbates and HPβCDs under different stress conditions. As shown in Figure 6, when subjected to autoclave for 5 cycles at 121 °C, 85% of PS 20, and 89% of PS 80 remained in the solution. When subjected to oxidation with 0.3% H_2_O_2_, both PS 20 and PS 80 suffered around 10% product loss within one week. Similarly, exposure to the light condition as described above resulted in the remaining of 94% and 90% for PS 20 and PS 80, respectively. In contrast to polysorbates, all KLEPTOSE^®^ HPβCDs remained stable when subjected to autoclave, light, and oxidative stresses. Statistical analysis of total concentration showed no significant difference between stressed KLEPTOSE^®^ HPβCDs and unstressed samples under those four stress conditions (the difference of autoclaved KLEPTOSEβ HPB from unstressed sample may result from positive error contributed by water loss during autoclave), suggesting that KLEPTOSE® HPβCDs showed good stability against different stresses in this study. Moreover, PS 20 was susceptible to light and autoclaving, and PS 80 was susceptible to light and oxidation.

**Figure 7:**
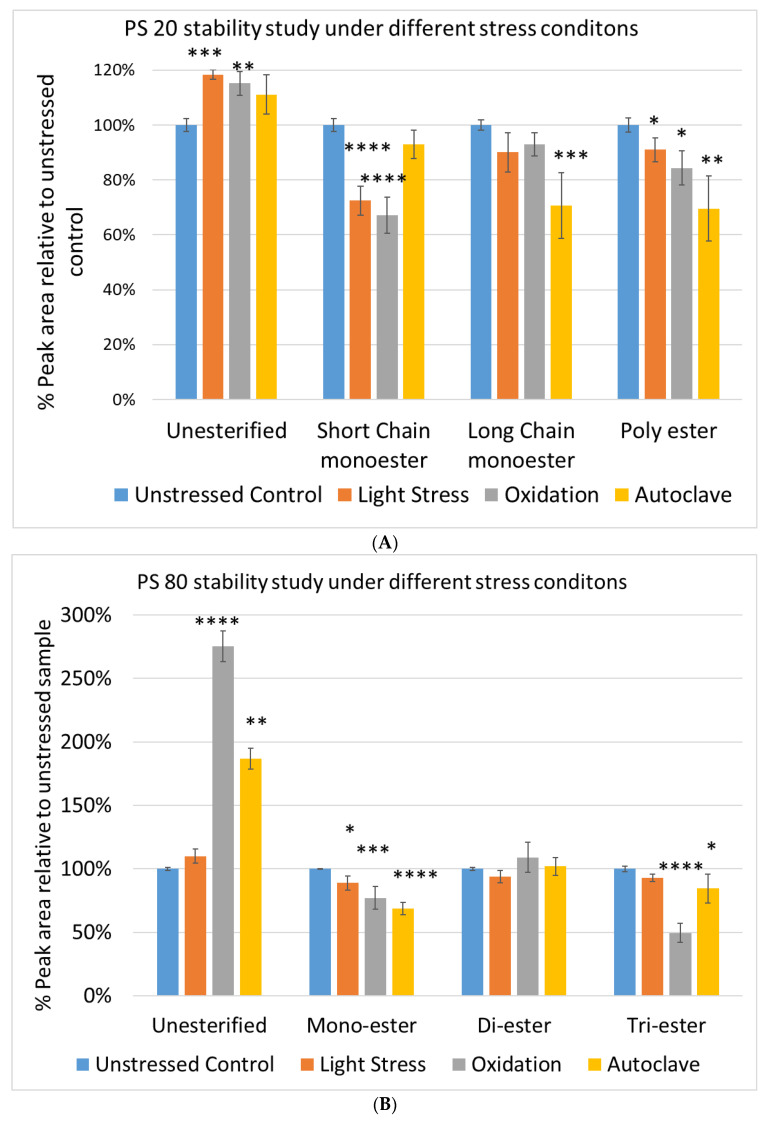

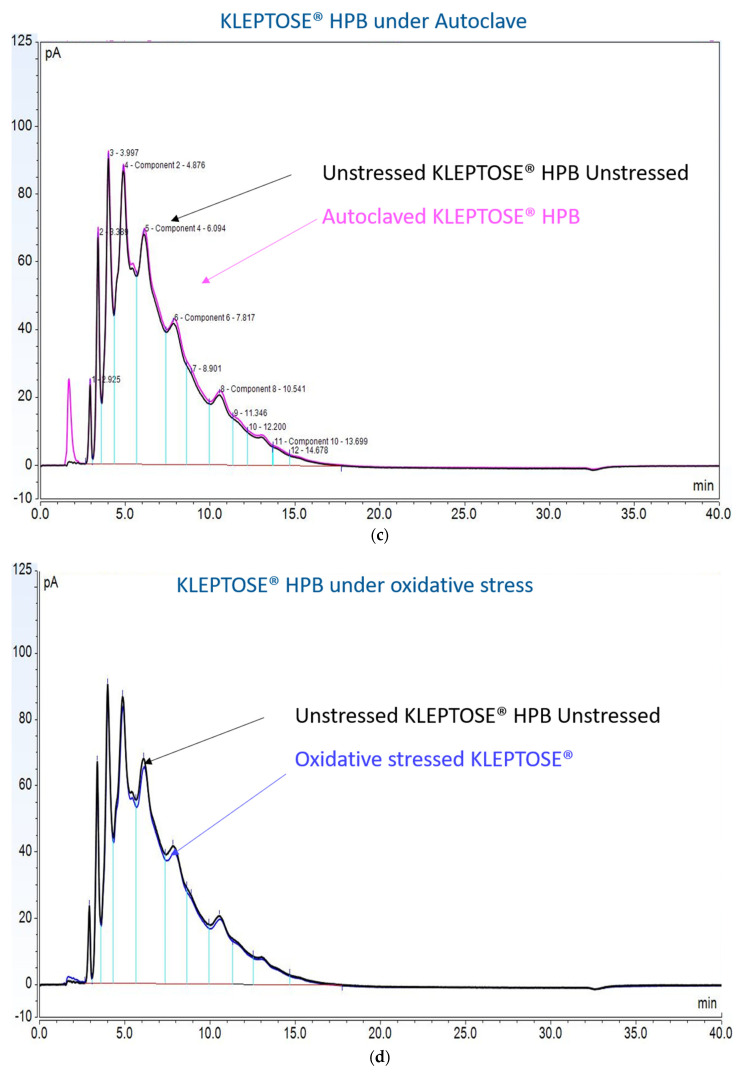

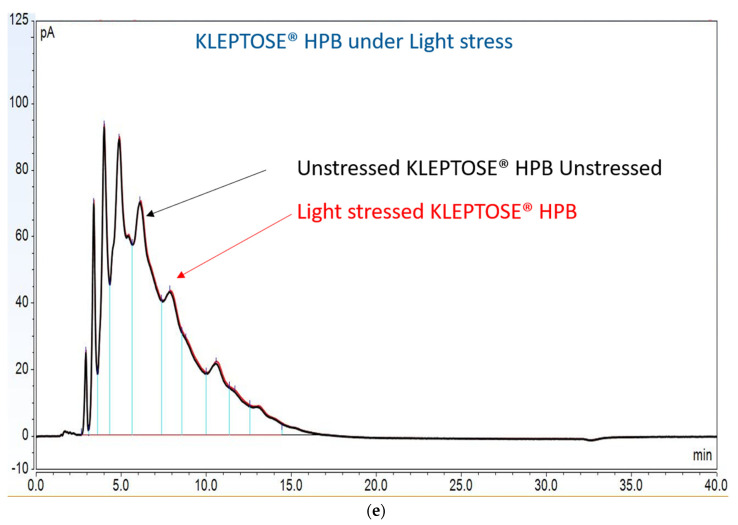
The degradation of POE sorbitan esters in PS 20 (**A**) and PS 80 (**B**); Profiling characterization KLEPTOSE® HPB under autoclave (**C**), oxidative (**D**), and light stresses (**E**). * *p* < 0.05, ** *p* < 0.01, *** *p* < 0.001, and **** *p* < 0.0001.

Again, we examined the chemical components of polysorbates and KLEPTOSE^®^ HPβCDs and monitored the change in their corresponding degradants under different stress conditions (Figure 7A,B). When subjected to those three stress conditions (autoclave, oxidative, and light stress), all major components in PS 20 (short/long EO chain esters and polyesters) and PS 80 (mono-, di-, and tri-esters) decreased, while non-esterified components significantly increased compared to those in unstressed samples. Statistical analysis of POE sorbitan esters showed that monoesters and polyesters underwent significant degradation with the buildup of unesterified components.

Furthermore, three KLEPTOSE^®^ HPβCDs were separated and analyzed on a CD screen column coupled with CAD detection. Representative LC–CAD chromatograms of unstressed and stressed KLEPTOSE^®^ HPB are shown in Figure 7C–E. For each KLEPTOSE^®^ HPβCD, the well-overlaid chromatograms of unstressed and stressed samples indicate that there is no significant degradation observed for all substituents under the autoclave, oxidative, and light stresses. 

### 2.3. Stabilization Benefits on Monoclonal Antibody

To compare the impact of polysorbate and KLEPTOSE^®^ HPβCD on protein stability, the physicochemical stability of monoclonal antibodies in formulations with PS 80 or KLEPTOSE^®^ HPB as stabilizers was investigated using adalimumab antibody as a model protein. As such, a series of stability tests using different stressors including mechanical (stirring), thermal, and light were carried out to induce particle formation and adalimumab degradation. Stress-induced particle formation and adalimumab degradation profiles were then measured and analyzed by different analytical strategies. 

#### 2.3.1. Subvisible Particle Formation of Adalimumab under Various Stresses

**Figure 8:**
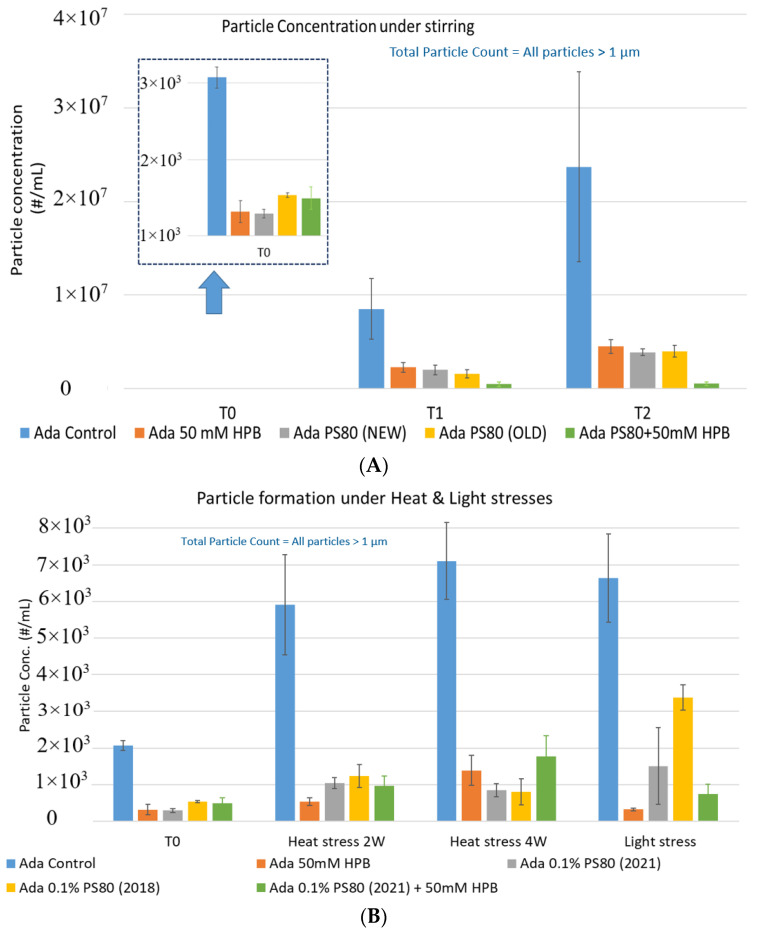
Particle formation with adalimumab in different formulations under stirring (**A**) and thermal and light stress (**B**).

Within 2 hours, stirring led to a significant increase in subvisible particle formation in all formulations (shown in Figure 8A) compared to unstressed samples (T0). Compared to the control formulation, the presence of either 50 mM KLEPTOSE^®^ HPB or 0.1% *w*/*w* PS 80 was able to reduce the formation of particles significantly. The extent of this reduction was comparable for all three formulations, suggesting that KLEPTOSE^®^ HPB was as effective as polysorbate 80 in preventing agitation stress induced particle formation. Notably, the performance of the aged PS 80 (made in 2018) was as efficient as the newer batch (made in 2021). The formulation containing the aged PS 80 (2018) produced only a slightly higher particle count compared to the newer batch of 2021. This can possibly be attributed to its higher peroxide value that caused a greater extent of protein oxidation. Interestingly, when PS 80 was used in combination with KLEPTOSE^®^ HPB, the formulation prevented further particle formation (down to 10% of particles formed in control buffer). This result may suggest an added benefit in using a combination of PS 80 and KLEPTOSE^®^ HPB to modulate particle formation in formulations.

As shown in Figure 8B, when subjected to heat stress at 40 °C / 75% R.H., a modest increase of particles in control buffer was formed over time during the 4-week incubation period. Adalimumab formulation in control buffer generated the highest number of subvisible particles. By contrast, the presence of KLEPTOSE^®^HPB in the formulation significantly reduced the formation of particles, and they offered similar protection as PS 80 against particle formation. 

Similarly, when subjected to light stress, the adalimumab formulation in control buffer produced the highest subvisible particles among all formulations. It was noted that both KLEPTOSE^®^ HPB-containing formulations prevented more particles formation as compared to PS80 formulations, suggesting that KLEPTOSE^®^ HPB can offer better protection from light-induced particle formation. Interestingly, unlike in the agitation study, the formulation containing the aged PS 80 produced a much higher particle count compared to the newer batch of PS80 when exposed to light stress. The particle sizes of the formulations were also analyzed on the Zetasizer Nano ZS to investigate the distribution profile of particles having a size range < 1 µm (data provided in Supporting Information). Similar observations were made whereby the presence of KLEPTOSE^®^ HPB resulted in enhanced formulation stability for either elevated thermal stress or light stress.

#### 2.3.2. Adalimumab Aggregation and Fragmentation Profiles under Various Stresses

**Figure 9:**
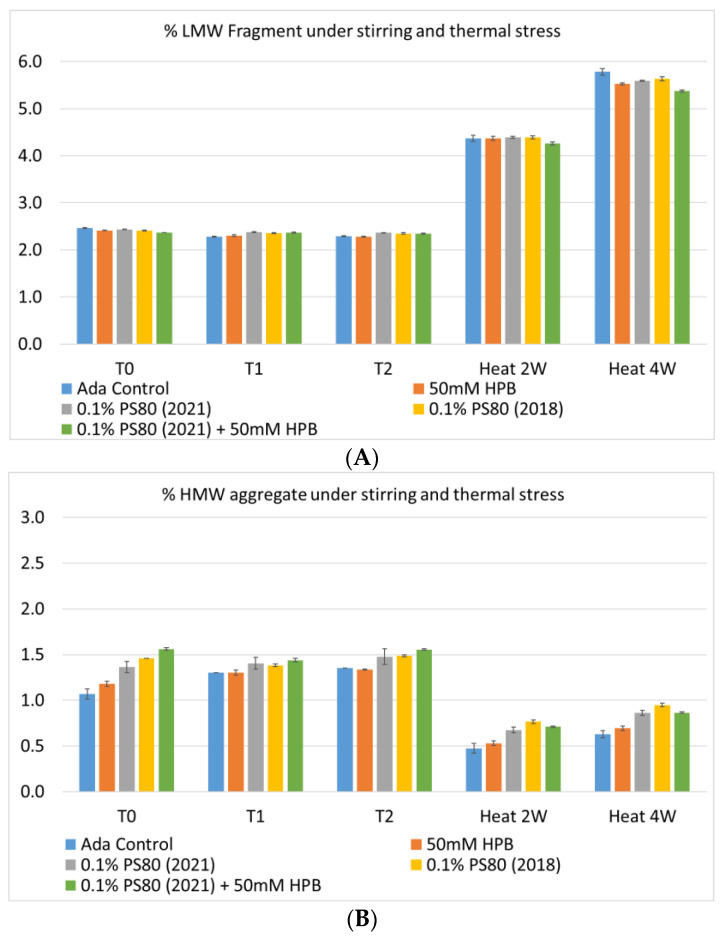

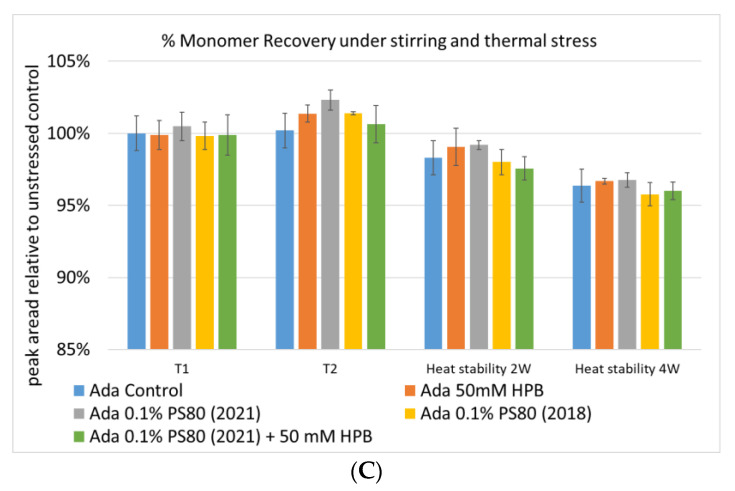
Fragmentation (**A**), aggregation (**B**), and monomer recovery (**C**) of adalimumab in different formulations under agitation in 2 hours and thermal stresses within 4 weeks.

Furthermore, the stability of adalimumab in five formulations under different stress conditions was assessed by SEC−HPLC by measuring aggregation and fragmentation in the formulations. Under agitation stress, as shown in Figure 9, adalimumab formulations in 1 hour (T1) and 2 hours (T2) showed slightly increased aggregation (high molecular weight or HMW species), but no change in fragmentation (low molecular weight or LMW species) level was observed in all five formulations. Similarly, monomer and total protein recovery (data not shown) in all five samples in this study showed comparable recovery relative to that of the adalimumab control formulation.

**Figure 10:**
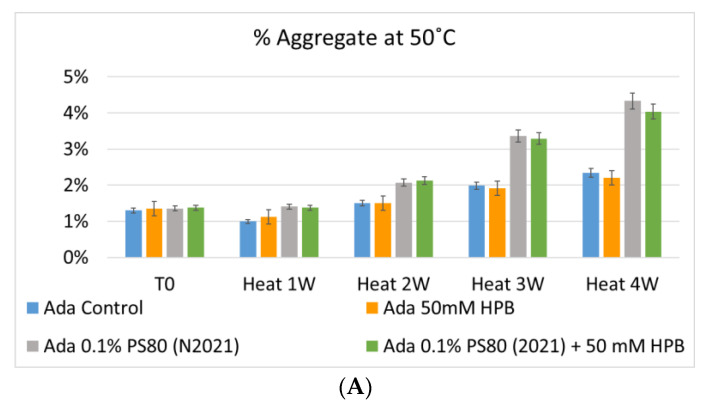

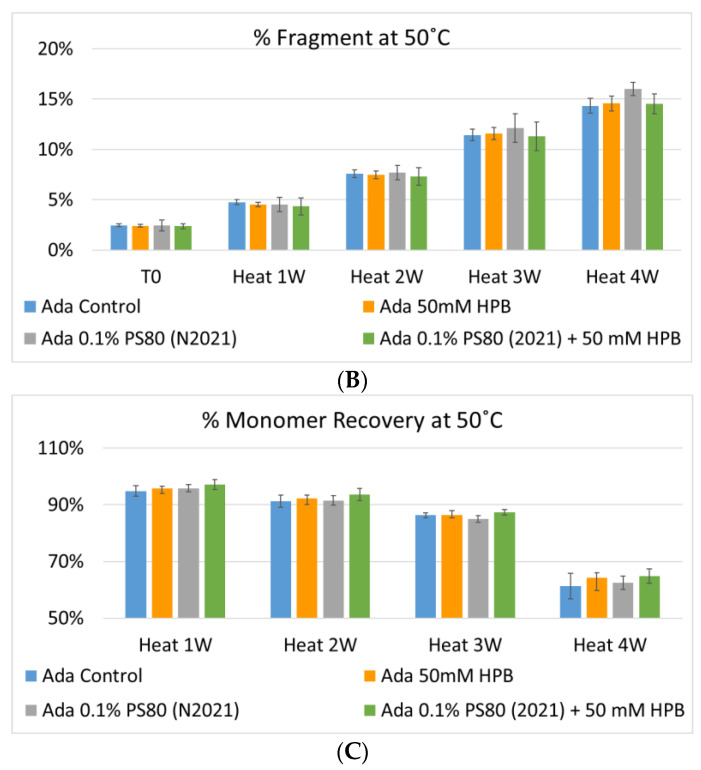
Aggregation (**A**), fragmentation (**B**) and monomer recovery (**C**) of adalimumab in different formulations under elevated thermal stresses (50 °C).

The adalimumab stability under thermal stress at 40 °C / 75% RH was monitored for 4 weeks. As shown in Figure 9, the %LMW fragmentation level increased by approximately 120% (from 2.5% to 5.5%) in all stressed samples compared to the unstressed control. At the same time, slightly lower %HMW aggregation levels in all stressed formulations were found (Figure 9B), which may have resulted from insoluble aggregates being removed before HPLC column separation. Additionally, monomer recovery in all formulations slightly decreased. However, there were no significant differences in aggregation and fragmentation levels in all five formulations, including adalimumab control formulations at different time points. Then, we examined the thermal stress effect at elevated temperatures of 50 °C to evaluate adalimumab stability (Figure 10). Under higher thermal stress, the KLEPTOSE^®^ HPB-containing formulation presented a significantly lower aggregation level (2.2%) compared to the polysorbate 80 formulation (4.3%). The KLEPTOSE^®^ HPB-containing formulation also showed a lower fragment level (1.9% vs. 3.6%) and higher monomer recovery (64.1% vs. 62.5%) compared to PS 80 formulations, while the combination of the PS 80 and KLEPTOSE^®^ HPB formulations obtained an intermediate value (3.4% for fragment percent, and 84.2% for monomer recovery). 

**Figure 11:**
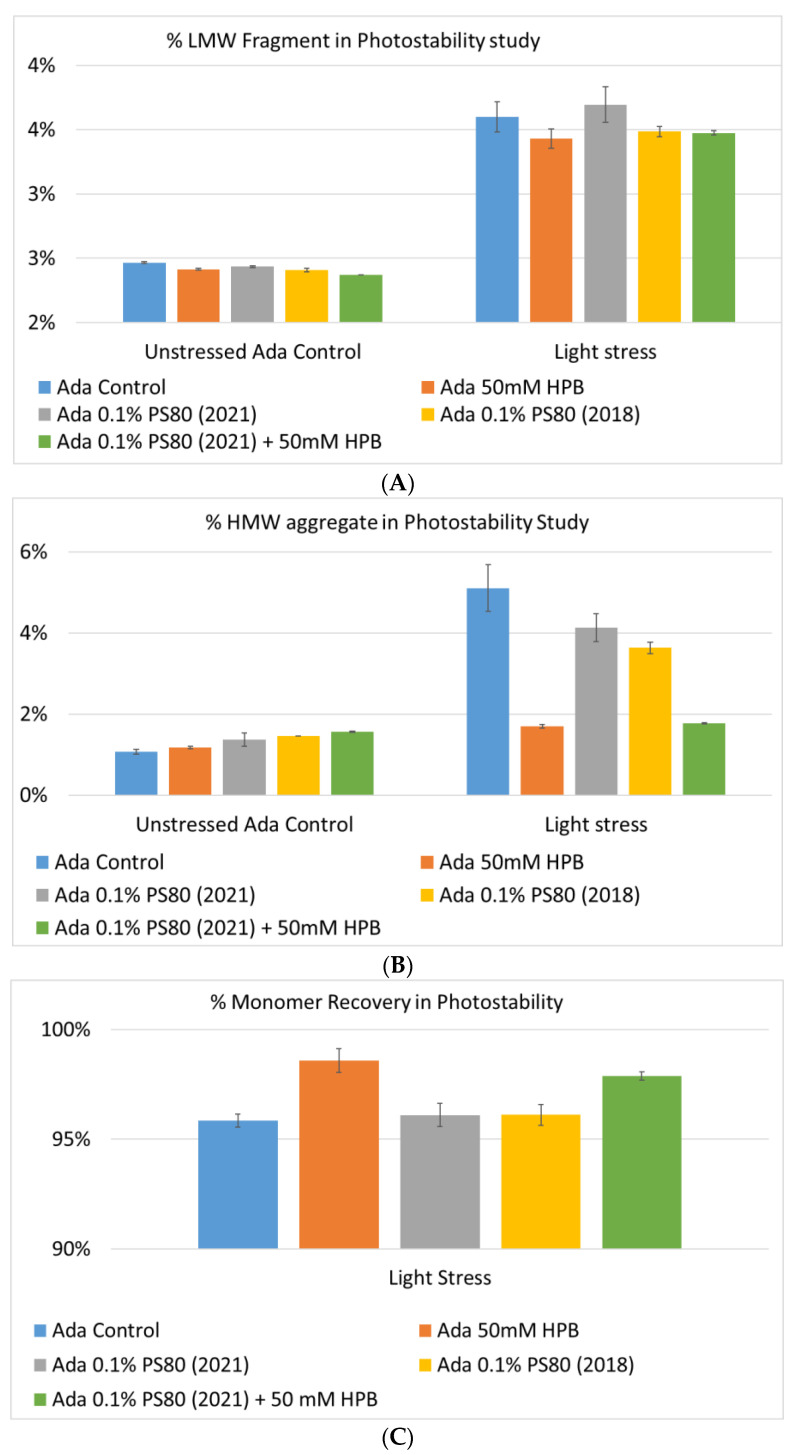
Aggregation (**A**), fragmentation (**B**) and monomer recovery (**C**) of adalimumab in different formulations under light stress.

Lastly, the photostability of adalimumab in five different formulations after exposure to ICH lighting conditions was investigated. As shown in Figure 11, light stress led to significant protein aggregation in control buffer as well as in PS 80-containing buffers in which %HMW increased from 1.07% to 5.11% for the control sample, from 1.18% to 4.13% for 0.1% PS 80 (2021), and from 1.46% to 3.63% for antibody 0.1% PS 80 (2018), respectively. Notably, two KLEPTOSE^®^ HPB formulations (KLEPTOSE^®^ HPB and KLEPTOSE^®^ HPB plus PS 80) presented relatively lower protein aggregation than the other formulations. Similar results were observed for adalimumab fragmentation under light stress in which %LMW of adalimumab in 50 mM KLEPTOSE^®^ HPB was the lowest among all formulations. In terms of monomer loss under light stress, both adalimumab samples in KLEPTOSE^®^ HPB formulations gained better recovery (98.6% and 97.9%, respectively) than the other three formulations, with around 96% recovery. Taken together, the presence of KLEPTOSE^®^ HPB in the sample protected adalimumab antibody from degradation and reduced the aggregation level compared to control buffer and PS 80 formulations.

#### 2.3.3. Adalimumab Charge Variant Profiles under Various Stresses

**Figure 12:**
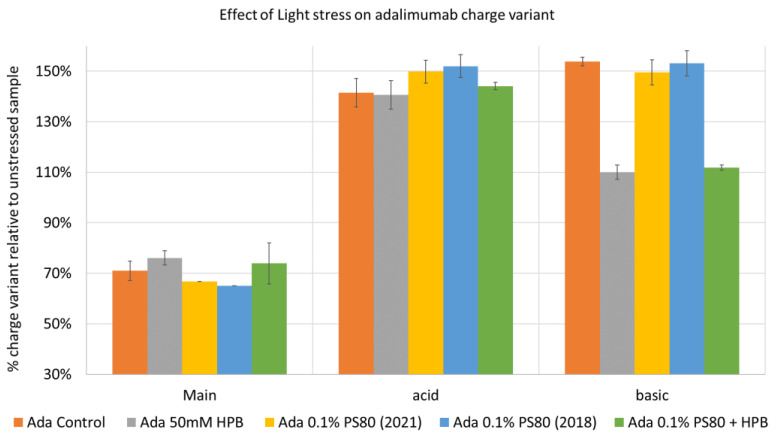
Adalimumab charge variants profiling under light stress.

Additionally, cation-exchange chromatography (CEX) using a salt gradient was deployed to characterize adalimumab antibody charge heterogeneity under thermal and light stress in different formulations. As shown in Supplementary Figure S4, when exposed to thermal stress within a 4-week period, acid variant peaks in all five adalimumab formulations showed a substantial increase up to approximately 140% compared to the unstressed sample with prolonged exposure time, whereas basic variant peaks started to increase after 2 weeks of thermal stress and reached up to around 110% after 4 weeks incubation. At the same time, the main peak decreased dramatically to 75% of the unstressed sample, which indicates the occurrence of important conformational changes of the antibody under thermal stress. When comparing the adalimumab charge variant profiles among formulations at the same time point, KLEPTOSE^®^ HPB-containing formulations exhibited the lowest change of both acid variants and basic variants, while maintaining the highest main peaks recovery in a 4-week period. When exposed to light stress (Figure 12), acidic peaks in three formulations (control buffer, KLEPTOSE^®^ HPB, and KLEPTOSE^®^ HPB/PS 80) increased to approximately 141%, while the values in both PS 80 formulations were up to 152% compared to the unstressed sample. Basic peaks in the three formulations without KLEPTOSE^®^ HPB (control buffer and both PS 80 formulations) showed a dramatic increase up to 154%, while the other two formulations containing KLEPTOSE^®^ HPB exhibited only a slightly higher value (110%) compared to the value in unstressed formulations. Similarly, main peaks in those three formulations without KLEPTOSE^®^ HPB suffered a significant loss (67% for PS 80 (2021), 65% for PS 80 (2018), and 71% for control buffer, respectively), while the main peak in both KLEPTOSE^®^ HPB-containing formulations showed lower losses (76% for the KLEPTOSE^®^ HPB formulation and 74% for the KLEPTOSE^®^ HPB/PS 80 formulation). 

### 2.4. Discussion

Adalimumab, a fully human recombinant immunoglobulin G1 (IgG1) anti-TNF monoclonal antibody, was first approved for the treatment of rheumatoid arthritis and subsequently expanded in the treatment of a wide variety of inflammatory conditions such as rheumatoid arthritis and Crohn’s disease [33]. Polysorbate 80 is optimized to minimize the formation of aggregates, as well as subvisible and visible particles during agitated and freeze–thaw conditions [34]. Cyclodextrins, cyclic oligosaccharides composed of (α-1,4)-linked α-D glucopyranose units, can improve water solubility and enhance bioavailability of drugs. Even though cyclodextrins, especially HPβCD, have been widely used in tablets, aqueous parenteral solutions, nasal sprays, and eye drop solutions, a systematic comparison between HPβCD and polysorbates and their impacts on biopharmaceuticals in formulations is lacking. In this study, we also established analytical strategies to investigate the chemical stability of polysorbates and HPβCDs under different stress conditions.

Quantitative analysis of polysorbates and KLEPTOSE^®^ HPβCD revealed that KLEPTOSE^®^ HPβCD maintains its chemical stability against different stress, while polysorbate 20 and 80 showed significant degradation. In addition, profiling characterization and degradation analysis upon different stresses revealed that PS 20 and PS 80 underwent significant hydrolytic degradation and oxidation, while the chemical structures of HPβCDs remained intact. These results combined with quantitative data (Figure 2 and Figure 4, Figure 5, Figure 6 and Figure 7) all serve to demonstrate that all grades of KLEPTOSE^®^ HPβCD showed greater stability than both PS 20 and 80 under different stress conditions. Many studies have indicated that the first step in hydrolysis of HPβCD is ring opening, followed by the hydrolysis of glycosidic linkages in acyclic oligosaccharides. Both macrocyclic rings and glycosidic bonds in HPβCD are relatively stable in mildly acidic or alkaline media. By contrast, sorbitan fatty acid esters are major components in polysorbates and can be easily degraded via hydrolysis in either acidic or alkaline conditions. Whilst our comparative results align with those studies, we also extend our findings to evaluate the potential impacts on the stability of biotherapeutics.

To explore KLEPTOSE^®^ HPβCD as a potential excipient for biopharmaceutical formulations, different stress conditions were chosen to evaluate stability benefits from different excipients by inducing degradation and aggregation as well as particle formation: agitation, and thermal and light stress. The degradation of polysorbate leads to a buildup of non-esterified fatty acids, which are poorly water soluble, eventually resulting in particle formation in the formulations. Protein aggregates and other types of subvisible particles present within biopharmaceutical formulations may negatively impact drug safety and efficacy. Under agitation stress (stirring), the adalimumab formulation without polysorbates and KLEPTOSE^®^ HPB showed an extremely high subvisible particle formation, while both PS 80 and KLEPTOSE^®^ HPB suppressed the particle formation efficiently under stirring. The addition of KLEPTOSE^®^ HPB into the PS 80 formulation even further reduced the particle numbers compared to the PS 80 formulation only. When subjected to thermal and light stresses, adalimumab in the control buffer gained a modest number of particles, while both PS 80- and KLEPTOSE^®^ HPB-containing formulations exhibited much lower particle numbers. The data also revealed that KLEPTOSE^®^ HPB-containing formulations showed enhanced efficiency to prevent particle formation compared to PS 80-containing formulation when subjected to light stress. Taken together, under stirring, light, or thermal stresses, KLEPTOSE^®^ HPB-containing formulations showed comparable effectiveness to prevent subvisible particle formation to PS 80-containing formulations. 

In addition, to study the potential impact of the degradation of PS 80 on the stability of adalimumab in parenteral formulations, both new batch and old batch PS 80 were formulated to examine the difference in their capacity to stabilize adalimumab under different conditions. The chemical components and peroxide values of PS 80 from the year 2018 and 2021 were analyzed and are summarized in Supplementary Table S5. The aged PS 80 showed enhanced peroxide value and non-esterified components compared to newer batch PS 80 (0.85 mEq vs. 0.29 mEq for peroxide value, and 5.26 vs. 4.60 for non-esterified components, respectively). Under different stress conditions including agitation, thermal, and light stresses, adalimumab formulations containing PS 80 (2018) exhibited higher particle numbers and slightly lower monomer recovery, although some quality attributes showed no or little difference, such as aggregation and fragmentation level. The formation of a greater number of sub-visible particles in the aged PS 80 formulation may be attributed to its degradation and subsequent buildup of fatty acid during storage. 

Adalimumab aggregation (HMW) and fragmentation (LMW) in five formulations under agitation and thermal stress were also investigated. However, no significant difference in all five formulations were found when comparing the aggregation and fragmentation levels; this may be due to the high stability of adalimumab in the formulations. Furthermore, particle sizes (<1 µm) for all formulations under thermal and light stresses were analyzed on the Zetasizer Nano ZS (Supplementary Figures S1–S3). The intensity size distributions of adalimumab with various formulations did not show any changes before and after thermal stress, indicating that no significant antibody aggregates were formed during the 4-week thermal stress period (Supplementary Figure S1). All data obtained above pointed out the super stability of adalimumab; thus, the stability effect of formulation may not be observed. An elevated temperature of 70 °C was applied to the formulations in the Zetasizer to further investigate the possibility of aggregation under elevated thermal stress. The particle size distributions (Supplementary Figure S2) revealed a larger extent of particle size increase and polydispersity at 70 °C for the formulation with PS 80 as compared to that of KLEPTOSE^®^ HPB. Altogether, under more severe thermal conditions, the KLEPTOSE^®^ HPB-containing formulation showed enhanced adalimumab stability compared to the PS 80 formulation. On the other hand, under light stress (Supplementary Figure S3) particle size was found to increase for adalimumab in control buffer, indicating the presence of aggregation, while the formulation with KLEPTOSE^®^ HPB did not show any changes in the particle size distributions.

Lastly, exposure to light stress induced aggregation and fragmentation of adalimumab in the control buffer. KLEPTOSE^®^ HPB presented a significantly lower protein aggregation and fragmentation level than the other formulations (control buffer and PS 80 formulations). Additionally, KLEPTOSE^®^ HPB formulations obtained the highest monomer recovery among all formulations used in the photostability study. Furthermore, charge variants are often related to antibody stability since they are involved in numerous degradation processes, and the changes in charge profiling are found to have structural and functional implications. Our data showed that the main species decreased predominantly, while both acidic and basic variants increased under light stress, and the presence of KLEPTOSE^®^ HPB in the formulation efficiently reduced the main species loss and suppressed basic variants' enhancement. Taken together, KLEPTOSE^®^ HPB clearly exhibited adalimumab protection from light stress compared to the other formulations, including PS 80.

## 3. Materials and Methods

### 3.1. Materials

All chemicals and reagents were used as received without further purification. Polysorbate 20 and 80, sodium phosphate monobasic, sodium phosphate dibasic, sodium chloride, sodium citrate, and mannitol were obtained from Sigma-Aldrich (St. Louis, MO, USA). Acetonitrile and methanol (HPLC grade) were from J.T. Baker (Phillipsburg, NJ, USA). Formic acid (LC–MS grade) was from Thermo Fisher Scientific (Waltham, MA, USA). Three different hydroxypropyl substituted HPβCD products, namely, KLEPTOSE^®^ HP (biopharma grade), KLEPTOSE^®^ HPB (biopharma grade), and KLEPTOSE^®^ HPB-LB (parenteral grade), were used without purification (chemical information summarized in Table 1). The monoclonal antibody adalimumab was produced in Chinese hamster ovary (CHO) cells (SD-16) and purified in-house.

### 3.2. Methods

#### 3.2.1. Sample Preparation and Stability Studies Design

Five different formulations were prepared for these studies: 1% *w*/*w* polysorbate 20 (PS 20) in water (milli-Q); 1% *w*/*w* polysorbate 80 in water (milli-Q); and 100 mM each of KLEPTOSE® HP, KLEPTOSE^®^ HPB, and KLEPTOSE^®^ HPB-LB in water (milli-Q). All formulations were filtered using a 0.45 µm PFTE filter before usage. All samples were subjected to four different stress conditions, namely, thermal stress, auto-clave, light, and agitation, to evaluate their chemical stability. For the thermal stability study, all samples were placed in a stability chamber (C500L, Weiss Technik, Reiskirchen, Germany) for 24 weeks at 40 °C and 75% RH as per ICH guidelines for an accelerated study. Samples were withdrawn at 1 week, 3 weeks, 5 weeks, 7 weeks, 12 weeks, and 24 weeks, respectively, for analysis. Each sample was prepared in duplicate per round. Each sample was reprepared and tested for data validation. 

In order to examine stability under severe heat stress, samples were also subjected to a steam sterilizing autoclave for five cycles at 121 °C. Uracil (20 µg/mL), as the internal standard (IS), was added to all formulation before autoclaving to account for water loss in samples during autoclave stress. A standard curve of uracil from 2.5–50 µg/mL was plotted to calculate response factor. Each stressed sample was calculated and normalized by its response factor. At the end of each autoclave cycle, samples were allowed to cool down to ~60 °C before starting the next cycle. After five autoclave cycles, samples were equilibrated to room temperature in 4 °C before centrifugation at 5000 rpm for 5 min. 

Photostability studies were performed in a photostability chamber (Pharma 500-L, Weiss Technik) according to the International Council for Harmonization (ICH) Q1B (option 2) guidelines, in which samples were exposed to white light for 1.2 million Lux hours and UV light for 200-watt hours per square meter. 

Forced oxidation was carried out in 0.3% *w*/*w* hydrogen peroxide at room temperature for one week prior to analysis. All samples were prepared in duplicate, followed by a second round of sample preparation and tests to validate the collected data.

#### 3.2.2. High Performance Liquid Chromatography with Refractive Index (RI) and Charged Aerosol Detection (HPLC–CAD)

Quantitation of KLEPTOSE^®^ samples was done on an Agilent Technologies 1200 Series HPLC with a 1260 Infinity refractive index (RI) detector and a Phenomenex Kinetex HILIC LC column. Samples were diluted to an analytical concentration of 35 mg/mL with LCMS-grade water. First, 10 µL of sample was injected and eluted at 1 mL/min for 10 min. A solution containing acetonitrile and water in the ratio of 7:3 was used as the mobile phase. Characterization of KLEPTOSE^®^ HPβCD samples was done using a CD Screen 150 × 4 mm column. The samples were diluted to a concentration of 35 mg/mL. Then 10 µL of sample was injected and eluted at 1 mL/min for 40 min. Water (Mobile Phase A), acetonitrile (Mobile Phase B), and methanol (Mobile Phase C) were used in a gradient condition, as shown in Supplementary Table S1.

A Dionex Ultimate 3000 HPLC system coupled to a Corona charged aerosol detector and an ultraviolet/visible light detector was used for both quantitation and characterization of polysorbates as well as KLEPTOSE^®^ HPβCD. Separation and quantitation of polysorbate 20 and polysorbate 80 samples were performed on a Waters Oasis MAX column. Samples were diluted to a concentration of 50 ppm. Then 50 µL of sample was injected and eluted at a rate of 1 mL/min for 12 min. Water (Mobile Phase A) and isopropyl alcohol (Mobile Phase B) were used in a gradient condition, as shown in Supplementary Table S2.

The separation and characterization of PS 20 and degradants was carried out using a Waters Acquity Glycan BEH Amide column. Samples were diluted to a concentration of 0.1% *w*/*w*. Then 10 µL of sample was injected and eluted at a flow rate of 0.3 mL/min for 65 min. Next, 0.1% LC–MS-grade formic acid in water (Mobile Phase A) and 0.08% LC–MS grade formic acid in acetonitrile (Mobile Phase B) was used in a gradient (shown in Table S3). PS 20 components were identified by matching the peaks to their expected retention time and integrating the area under the respective peaks. Characterization of PS 80 samples was done using a Zorbax SB-AQ narrow bore RR column. Samples were diluted to a concentration of 0.1% *w*/*w*. Then 10 µL of sample was injected and eluted at a flow rate of 0.5 mL/min for 60 min. Next, 0.1% LC–MS grade formic acid in water (Mobile Phase A) and 0.08% LC–MS grade formic acid in acetonitrile (Mobile Phase B) were used in a gradient. PS 80 components were identified by matching the peaks to their expected retention time and integrating the area under the respective peaks.

#### 3.2.3. Liquid Chromatography–Mass Spectrometry (LC–MS)

KLEPTOSE^®^ HP, HPB, and HPB-LB, and polysorbates were separated and identified on the Waters Acquity H Class UPLC system with a QDa detector. Degradants of polysorbates and substituents of HPβCD (degree of substitution, the average number of substituents on a cyclodextrin (CD) molecule) were identified by mass to charge (*m/z*) ratio. The QDa was operated in an electrospray positive ion mode by applying a voltage of 0.8 kV to the ESI capillary, and the cone voltage was set at 15 V. The desolvation temperature was set at 600 °C. A full mass spectrum between an *m/z* of 100 and 1250 was acquired at a sampling rate of 2.0 points/s. The analytical conditions were the same as those of the characterization analysis on the Dionex HPLC–CAD system. All samples were diluted to a suitable concentration before analysis. 

#### 3.2.4. SEC–HPLC

Stability of adalimumab in the various formulations was evaluated using a Waters Acquity HPLC with a XBridge BEH SEC column (200 Å, 3.5 µm, 7.8 × 300 mm). Samples were centrifuged at 21,000× *g* for 15 min at 4 °C to remove debris before HPLC analysis. First, 10 µL of each sample was injected and eluted isocratically at a flow rate of 0.5 mL/min. A solution of 25 mM sodium phosphate and 150 mM sodium chloride, pH 6.8, was used as the mobile phase. Absorbance was recorded at 280 nm, and relative amounts of monomer were obtained by integrating the area of the peaks. 

#### 3.2.5. Adalimumab Formulation Stability Studies

Protein formulations were prepared from the monoclonal antibody adalimumab produced in-house: (1) adalimumab in formulation buffer only (140 mM sodium phosphate, 73.5 mM of sodium citrate, 105 mM NaCl, pH 5.2), (2) adalimumab in formulation buffer with 50 mM KLEPTOSE^®^ HPB, (3) adalimumab in formulation buffer with 0.1% *w*/*w* PS 80 (2018: manufactured in Y2018), (4) adalimumab in formulation with 0.1% *w*/*w* PS 80 (2021: manufactured in Y2021), and (5) adalimumab in formulation buffer with 50 mM KLEPTOSE^®^ HPB and 0.1% *w*/*w* PS 80 (2021). All formulations were formulated at a protein concentration of 5 mg/mL. PS 80 batches from two different years were included in the formulations to study the impact of PS 80 aging on their protective efficiency. 

All adalimumab formulations were subjected to agitation stress (stirring at 200 rpm for 2 h), thermal stress at 40 °C/75% RH, and light stress as per ICH Q1B (option 2) in a photostability chamber for stability evaluation. 

#### 3.2.6. Determination of Peroxide Value

Peroxide determination was performed with a commercially available glucose (GO) assay kit (Sigma, St. Louis, MO, USA). Hydrogen peroxide reacts with o-dianisidine in the presence of peroxidase to form a pink-colored product, which absorbs strongly at 540 nm. The intensity measured at 540 nm is proportional to the concentration of peroxide in the formulation.

#### 3.2.7. Subvisible Particles Analysis by Micro-Flow Imaging (MFI)

Subvisible particles in the formulations were further assessed by MFI (MFI 5200, Protei Simple, San Jose, CA, USA) with a silane-coated 100 μm flow cell. Typically, samples may need to be diluted to suitable particle concentrations before testing if the concentration is too high. Particle concentration results were reported as cumulative particle counts per mL for ≥2, ≥5, ≥10, and ≥25 µm size ranges, as well as ≥5 µm nonspherical particles with an aspect ratio of <0.70.

#### 3.2.8. Antibody Particle Size Analysis

Smaller particles size (less than 1 µm) were also measured with DLS on a Zetasizer Nano ZS (Malvern Panalytical, Malvern, UK) to evaluate the adalimumab particle size stability in different formulations. Three successive measurements were conducted per sample after 60 s of equilibration in the measurement cell. Intensity particle size distributions were used for comparison to reduce the assumptions on particle shape and optical properties during conversion to volume distributions. 

#### 3.2.9. Cation-Exchange Chromatography (CEX) for Charge Heterogeneity Profiling of Adalimumab

Charge variants of adalimumab were separated and analyzed on a Waters Protein-Pak™ Hi Res WCX column (CM 7 µm, 4.6 × 100 mm) using a CEX-HPLC system. A fixed-pH (pH 6.8 with 25 mM of sodium phosphate) salt gradient method (0–75 mM of NaCl in 20 min) has been developed for CEX chromatographic separation. First, 5 µg of adalimumab per sample was injected for analysis. Acidic variants, main isoform, and basic variants were separated and calculated to characterize charge variants profiles. 

#### 3.2.10. Statistical Analysis

Statistical significance was determined by analysis of variance (ANOVA) in Minitab. A *p* value of < 0.05 indicated statistical significance: * *p* < 0.05, ** *p* < 0.01, *** *p* < 0.001, and **** *p* < 0.0001.

## 4. Conclusions

HPβCD has been applied in several approved parenteral small molecule drugs. In view of its high water solubility and extremely stable structure, HPβCD shows great promise as a multifunctional excipient to prevent protein aggregation and to reduce subvisible particle formation in biopharma downstream processes and formulation. To overcome formulation challenges with the use of polysorbates, significant effort has been made to find a more suitable polysorbate alternative. Among them, Poloxamer 188 remains the most promising alternative and has been successfully used in commercial formulation. However, a recent study reveals that Poloxamer 188 also suffers from significant degradation under different stress conditions, for example, the degradants are up to 46% when incubated with hydrogen peroxide and increases up to 38% when subjected to thermal stress at 40 °C for 24 weeks [35].

To our knowledge, there have not been any other studies directly comparing polysorbates and HPβCD regarding their chemical stability and benefits to protein stability when used in biopharmaceutical formulations. The data presented here strongly suggest that HPβCD exhibits excellent stability during various stress conditions. Furthermore, HPβCD has been shown to significantly protect the adalimumab antibody from light stress and reduce subvisible particle formation during agitation stress. These findings have proven HPβCD to be an effective alternative functional excipient to polysorbate in therapeutic protein formulations. 

Nevertheless, further studies to elucidate the stabilization mechanism of HPβCD on proteins are still warranted. Evaluation of biological activity and changes in the protein molecular level may offer insights into the protective function of HPβCD on proteins.

## Figures and Tables

**Table 1:** Key points of the KLEPTOSE® range of hydroxypropyl beta-cyclodextrins (HPβCDs).

Sample	KLEPTOSE® HP, Biopharma	KLEPTOSE® HPB, Biopharma	KLEPTOSE® HPB-LB, Parental
Molecular Weight (g/mol)	1501 (nominal)	1387 (nominal)	1338–1424
Molar Substitution (MS)	0.81–0.99 (0.9, nominal)	0.58–0.68 (0.62, nominal)	0.50–0.71
Degree of Substitution (DS)	6.7 (Nominal)	4.3 (Nominal)	NA
Solubility in H_2_O	>50% (20 °C, *w*/*w*%)		
Residual Beta-CD	0.10%	0.7–0.8%	0.30%

**Table 2:** LC/ESI–MS identification results of PS 20, PS 80, and HPBCD.

Sample	Group	Identified Components (MS Detection)
PS 20	Non-esterified components	Polyoxyethylene (POE), POE isosorbide, POE sorbitan
	Short ethylene oxide chain monoesters	(POE isosorbide, POE sorbitan) mono (laurate, myristate)
	Long ethylene oxide chain monoesters	(POE isosorbide, POE sorbitan) mono (palmitate, sterate)
	Polyesters (di- and tri-esters)	(POE isosorbide, POE sorbitan)-di, tri, or mixed (laurate, myristate)
PS 80	Non-esterified components	POE, POE isosorbide, POE sorbitan
	Monoesters	(POE isosorbide, POE sorbitan) mono (oleate, linoleate)
	Diesters	POE isosorbide and POE sorbitan dioleate
	Tri-esters	POE isosorbide and POE sorbitan trioleate
KLEPTOSE^®^ HPB	Beta-CD	Beta-CD
	HP-β-CD	HP-DS1-β-CD
		HP-DS2-β-CD
		HP-DS3-β-CD
		HP-DS4-β-CD
		HP-DS5-β-CD
		HP-DS6-β-CD
		HP-DS7-β-CD
		HP-DS8-β-CD
		HP-DS9-β-CD
		HP-DS10-β-CD

## Data Availability

The data presented in this study are available on request from the corresponding author.

## Supplementary Materials

The following supporting information can be downloaded at: https://www.mdpi.com/article/10.3390/molecules27196497/s1, Table S1. Mobile phase gradient table for KLEPTOSE® Characterization on HPLC-CAD. Table S2. Gradient table for Polysorbate Quantitation on HPLC-RI. Table S3. Gradient table for characterization 0.1% *w*/*w* Polysorbate 20 on HPLC-CAD. Table S4. Gradient table for characterization 0.1% *w*/*w* Polysorbate 80 on HPLC-CAD. Table S5. Summary of Polysorbate 20 and 80 chemical information. Figure S1 for thermal stressed sample at 40°C / 75% RH, Figure S2 for samples at elevated temperature at 25 °C and 70 °C and Figure S3 for light stressed samples. Figure S4. Charge Variants of adalimumab formulations under thermal and light stress conditions.

## Sample Availability

Samples of the compounds are not available from authors.
