# Supplementary material for: Polysorbates versus Hydroxypropyl Beta-Cyclodextrin (HPβCD): Comparative Study on Excipient Stability and Stabilization Benefits on Monoclonal Antibodies
Source: Molecules. 2022 Oct 1;27(19):6497. doi: 10.3390/molecules27196497 (PMC9572940; doi:10.3390/molecules27196497)
Supplement: Supplementary file 1 [file molecules-27-06497-s001.zip › molecules-1917043-supplementary.pdf]

Supplementary information

1. Supplementary Table Chromatographic gradient conditions for the HPLC analysis of Polysorbates and HPβCD

Table S1. Mobile phase gradient table for KLEPTOSE® Characterization on HPLC-CAD

**Time (min)**	**Mobile Phase Composition**			**Curve**
	**%A**	**%B**	**%C**	
-5.00	65	0	35	6
0.00	65	0	35	6
7.50	65	0	35	6
25.00	50	15	35	6
30.00	50	15	35	6
30.10	65	0	35	6
40.00	65	0	35	6

Table S2. Gradient table for Polysorbate Quantitation on HPLC-RI

**Time (min)**	**Flow (ml/min)**	**% A**	**% B**
0.0	1.0	80	20
4.0	1.0	80	20
4.1	1.0	0.0	100
9.0	1.0	80	20
9.1	1.0	80	20
12.0	1.0	80	20

Table S3. Gradient table for characterization 0.1% w/w Polysorbate 20 on HPLC-CAD

**Time (min)**	**Flow (ml/min)**	**% A**	**% B**
0.0	0.3	98	2
2.0	0.3	98	2
37	0.3	5	95
42	0.3	5	95
47	0.3	0	100

55	0.3	0	100
58	0.3	98	2
65	0.3	98	2

Table S4. Gradient table for characterization 0.1% w/w Polysorbate 80 on HPLC-CAD

Time (min)	Flow (ml/min)	% A	% B
0.0	0.5	98.0	2.0
2.0	0.5	98.0	2.0
30.0	0.5	25.0	75.0
38.0	0.5	4.0	96.0
43.0	0.5	4.0	96.0
45.0	0.5	0.0	100.0
50.0	0.5	0.0	100.0
52.0	0.5	98.0	2.0
60.0	0.5	98.0	2.0

2. **Table S5.** Summary of Polysorbate 20 and 80 chemical information

sample	non-esterified components	Mono-esters	Di-esters	Tri-esters	peroxide value (mEq)
PS 80 (2018)	5.26	22.05	29.53	16.88	0.85
PS 80 (2021)	4.60	21.64	29.23	17.60	0.29

3. Supplementary Particle size distribution analysis of adalimumab formulations under different stress conditions (Figure S2 for thermal stressed sample at 40˚C / 75% RH, figure S1 for samples at elevated temperature at 25 ˚C and 70 ˚C, figure S3 for light stressed samples)

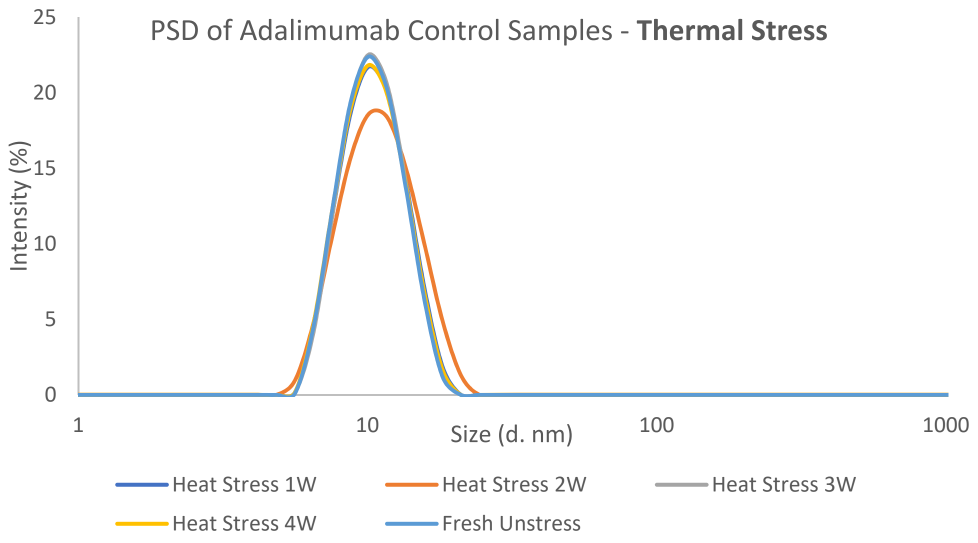

Supplementary Figure **S1.1** Overlay particle size distribution of adalimumab in citric phosphate buffer only for a period of 4 weeks thermal stress at 40˚C / 75% RH. No significant changes observed from the size measurements due to heat stress, difference in particle size for week 2 is related to inherent variability noted in samples with neat adalimumab.

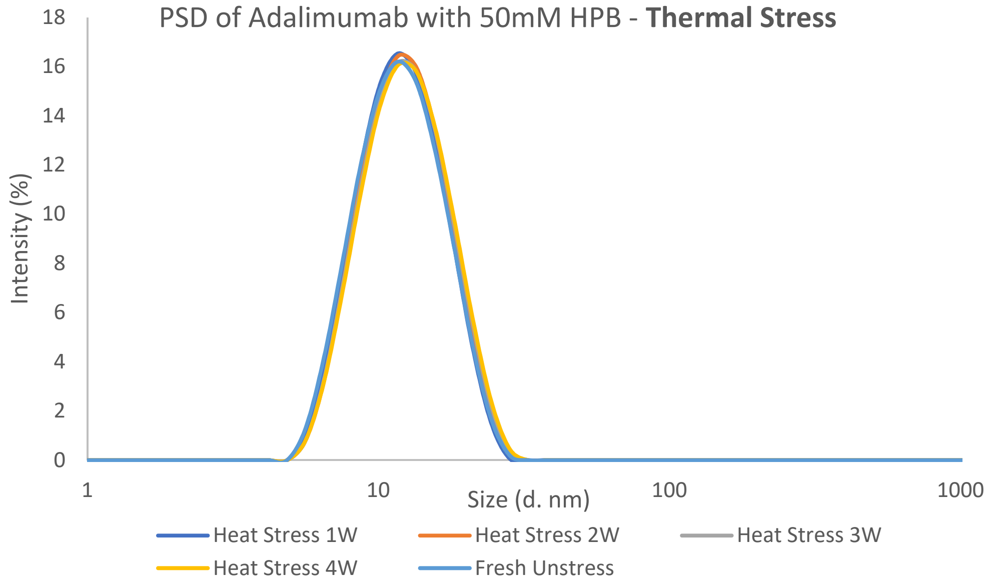

Supplementary **Figure S1.2** Overlay particle size distribution of adalimumab with 50mM HPB for a period of 4 weeks thermal stress at 40˚C / 75% RH. No significant changes observed from the size measurements.

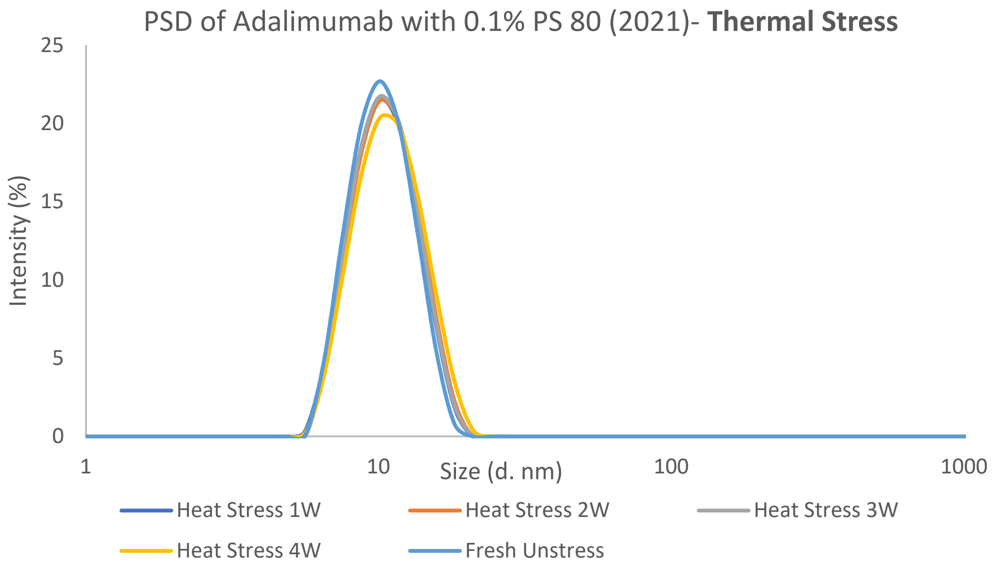

Supplementary **Figure S1.3** Overlay particle size distribution of adalimumab with 0.1% PS 80 (2021) for a period of 4 weeks thermal stress at 40˚C / 75% RH. No significant changes observed from the size measurements.

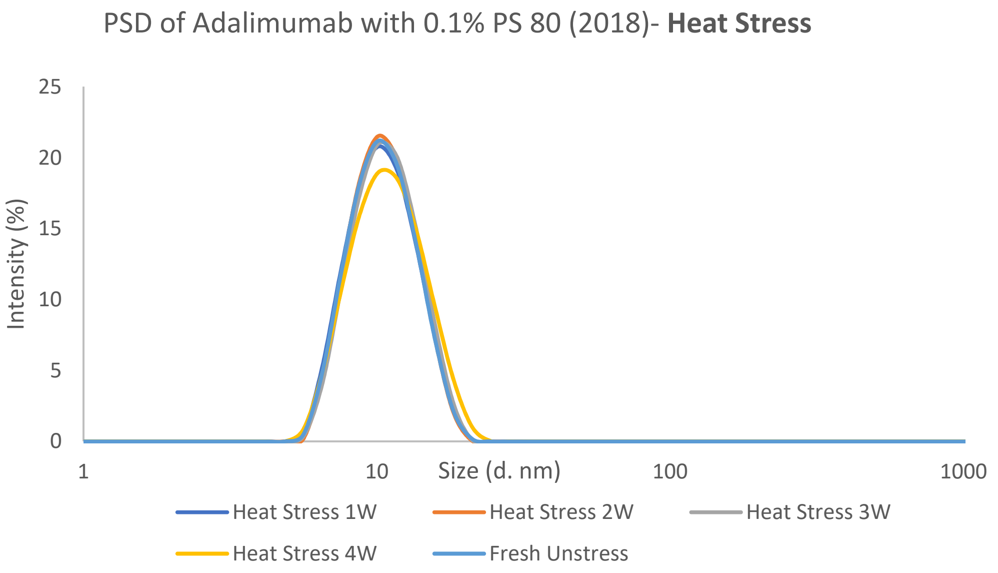

Supplementary **Figure S1.4** Overlay particle size distribution of adalimumab with 0.1% PS80 (2018) for a period of 4 weeks thermal stress at 40˚C / 75% RH. Slight increase in particle size and polydispersity index observed for the 4 weeks sample.

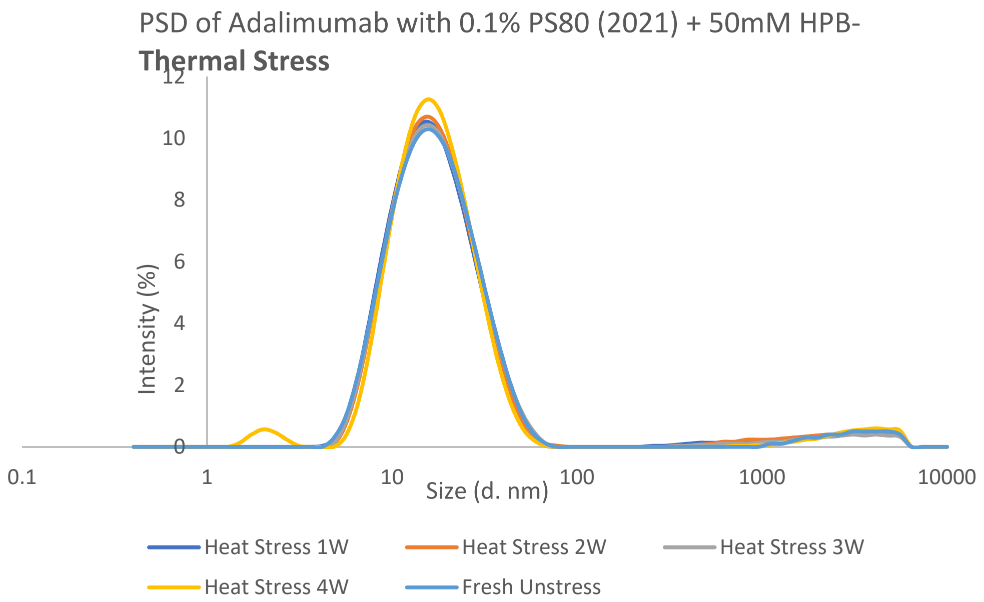

Supplementary **Figure S1.5** Overlay particle size distribution of adalimumab with 0.1% PS80 (2021) + 50mM HPB for a period of 4 weeks thermal stress at 40˚C / 75% RH. No significant changes observed from the size measurements.

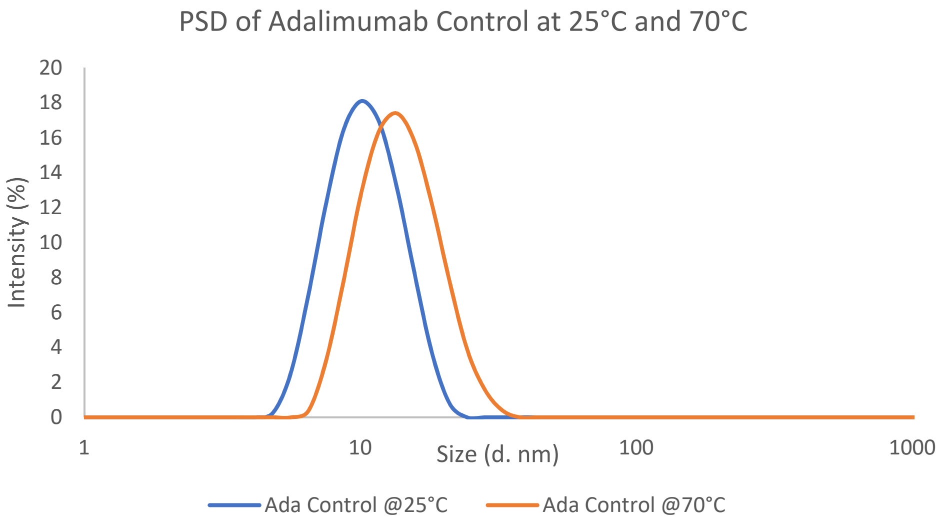

Supplementary **Figure S2.1** Overlay particle size distribution of adalimumab citric phosphate buffer only analyzed at 25°C and 70°C in Zetasizer Nano. Results shows that there is an increase of 33% in the Z-avg size with no increase in the polydispersity.

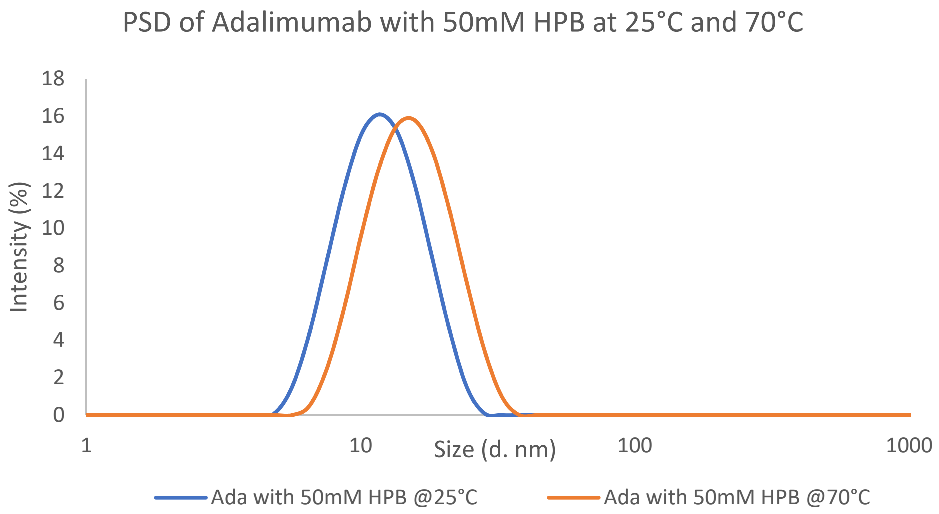

Supplementary **Figure S2.2** Overlay particle size distribution of adalimumab with 50mM HPB analyzed at 25°C and 70°C in Zetasizer Nano. Results shows that there is an increase of 31% in the Z-avg size with no increase in the polydispersity.

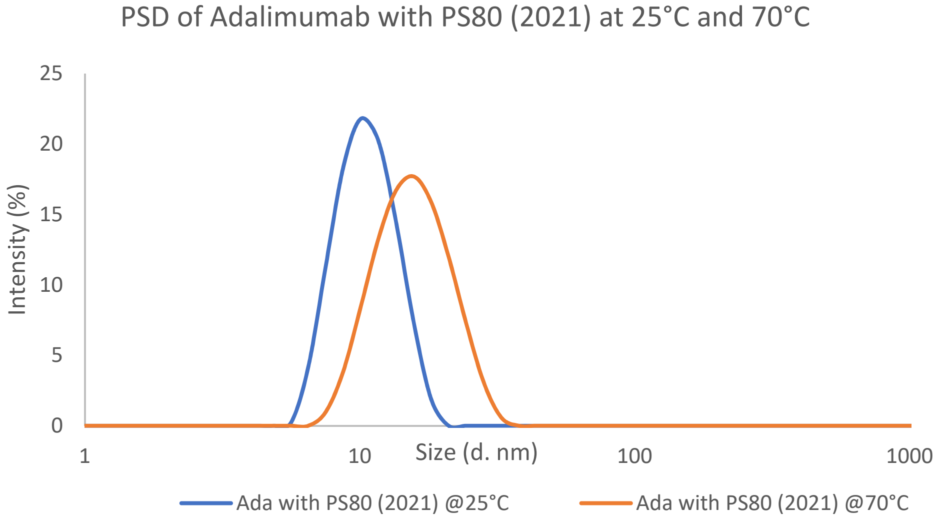

Supplementary **Figure S2.3** Overlay particle size distribution of adalimumab with PS80 (2021) analyzed at 25°C and 70°C in Zetasizer Nano. Results shows that there is an increase of 44% in the Z-avg size coupled with an increase of 150% in the polydispersity.

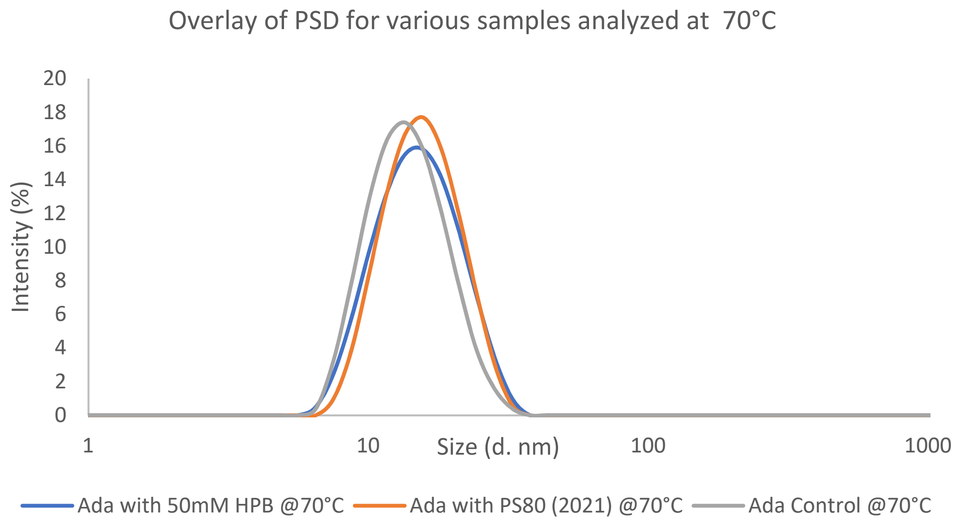

Supplementary **Figure S2.4** Overlay particle size distribution of adalimumab in buffer only, with 50mM HPB and with PS80 (2021) analyzed 70°C in Zetasizer Nano. Particle size of HPB sample is similar to the other 2 samples as the particle size of adalimumab with HPB was slightly larger at 25°C.

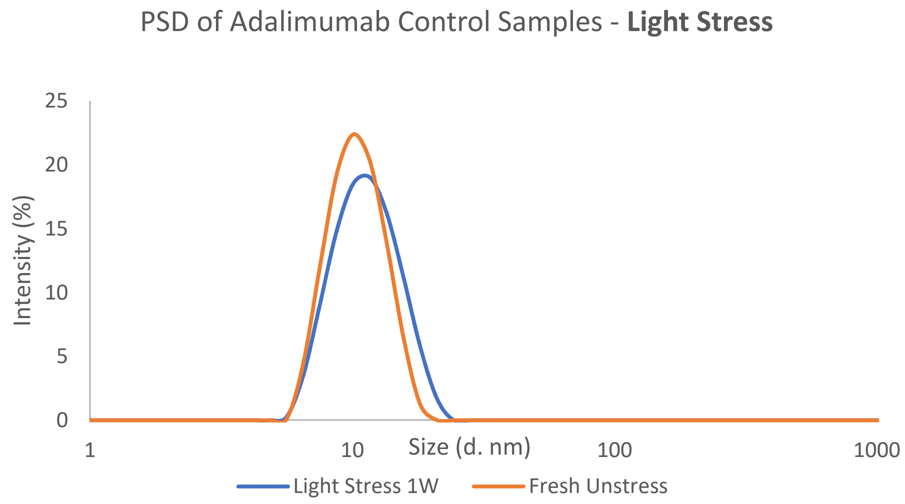

Supplementary **Figure S3.1** Overlay particle size distribution of adalimumab in citric phosphate buffer only under light stress. Increase in the Z-avg and polydispersity was observed.

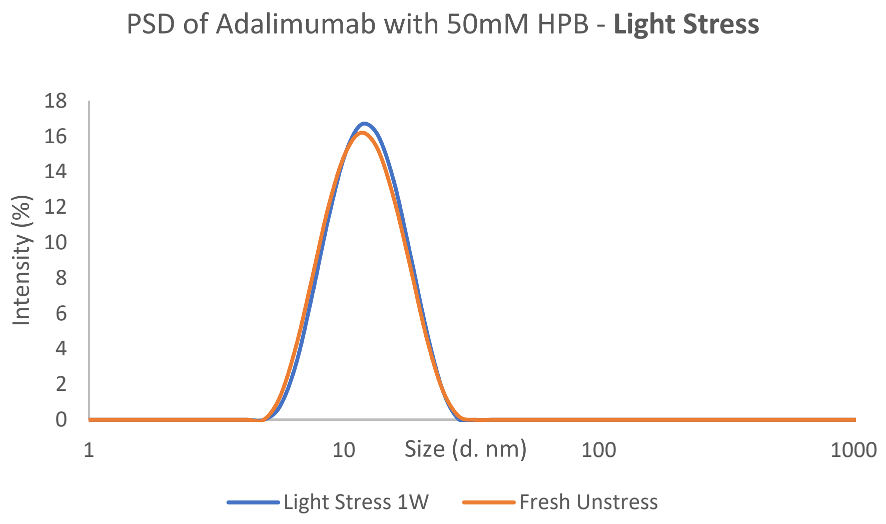

Supplementary **Figure S3.2** Overlay particle size distribution of adalimumab with 50mM HPB for a period of 1 week under light stress. No significant increase in the Z-avg and polydispersity was observed.

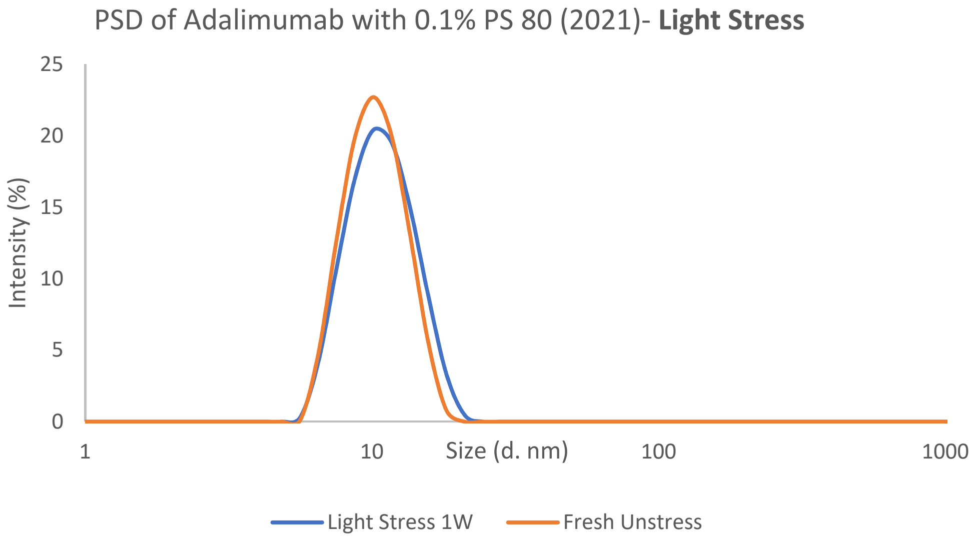

Supplementary **Figure S3.3** Overlay particle size distribution of adalimumab with 0.1% PS80 (2021) under light stress. Slight increase in the Z-avg and polydispersity was observed.

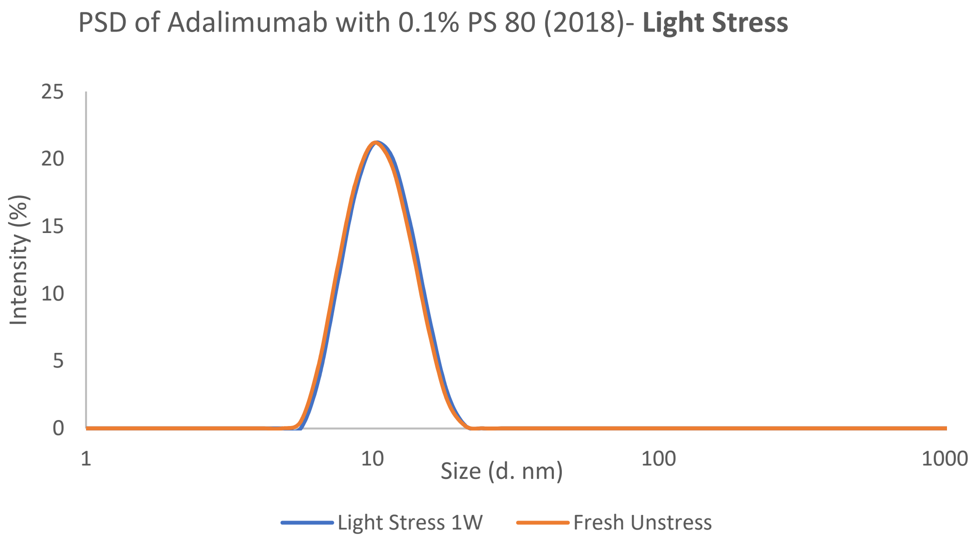

Supplementary **Figure S3.4** Overlay particle size distribution of adalimumab with 0.1% PS80 (2018) under light stress. No significant increase in the Z-avg and polydispersity was observed.

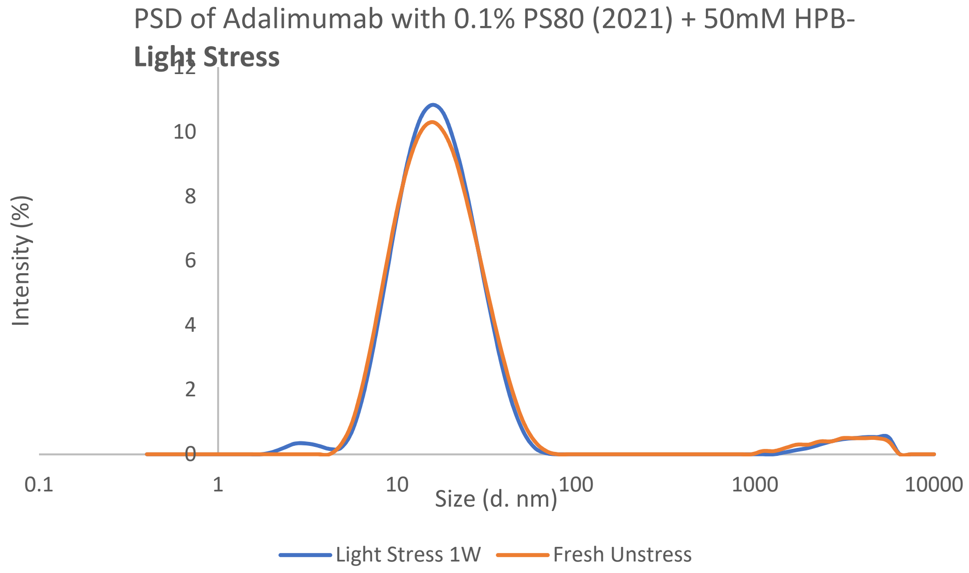

Supplementary **Figure S3.5** Overlay particle size distribution of adalimumab with 0.1% PS80 (2021) + 50mM HPB under light stress. Appearance of small particle size population was observed.

4. Charge Variants of adalimumab formulations under thermal and light stress conditions

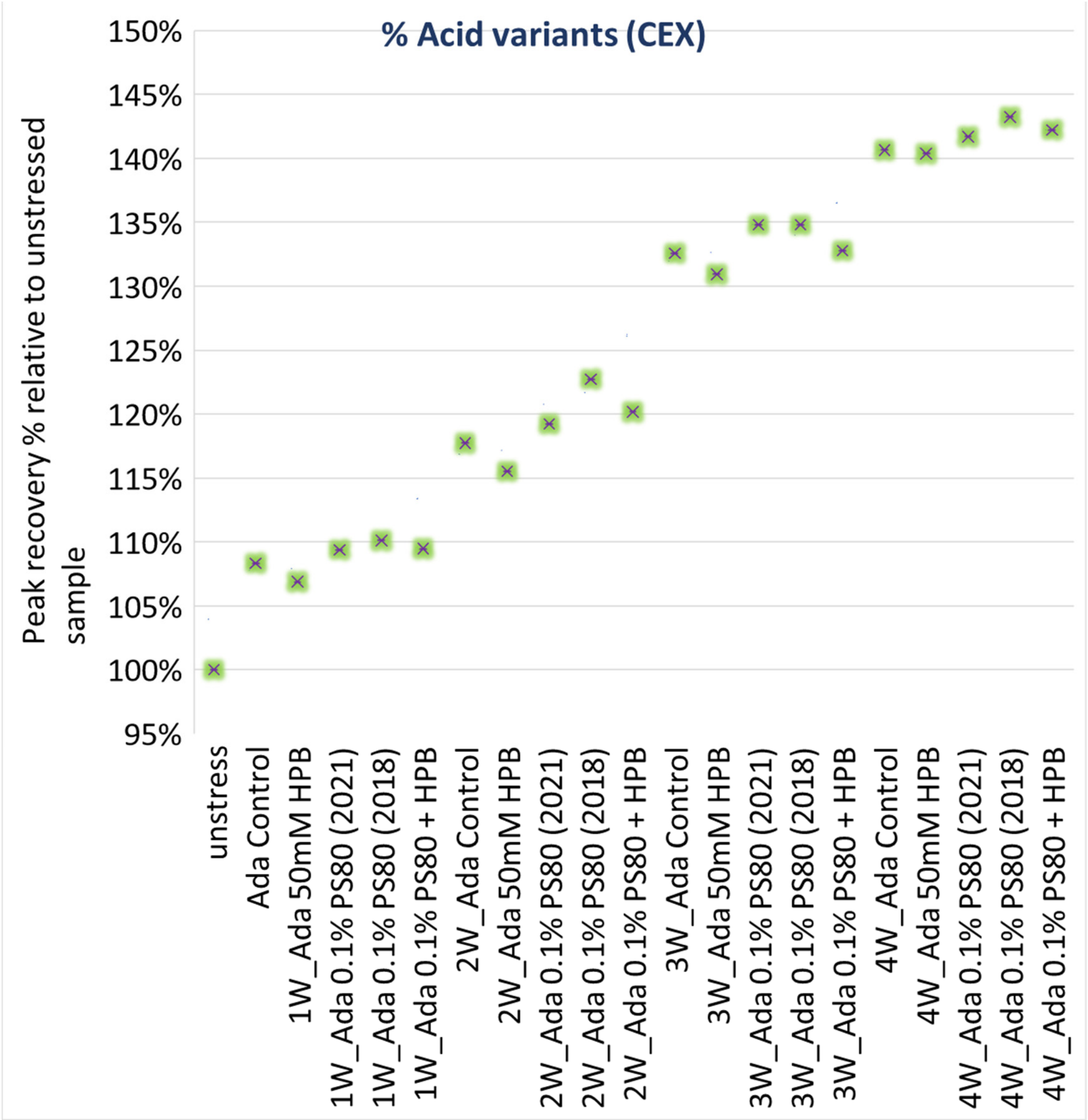

Supplementary Figure S4.1 Acid Species of adalimumab formulations under thermal and light stress conditions.

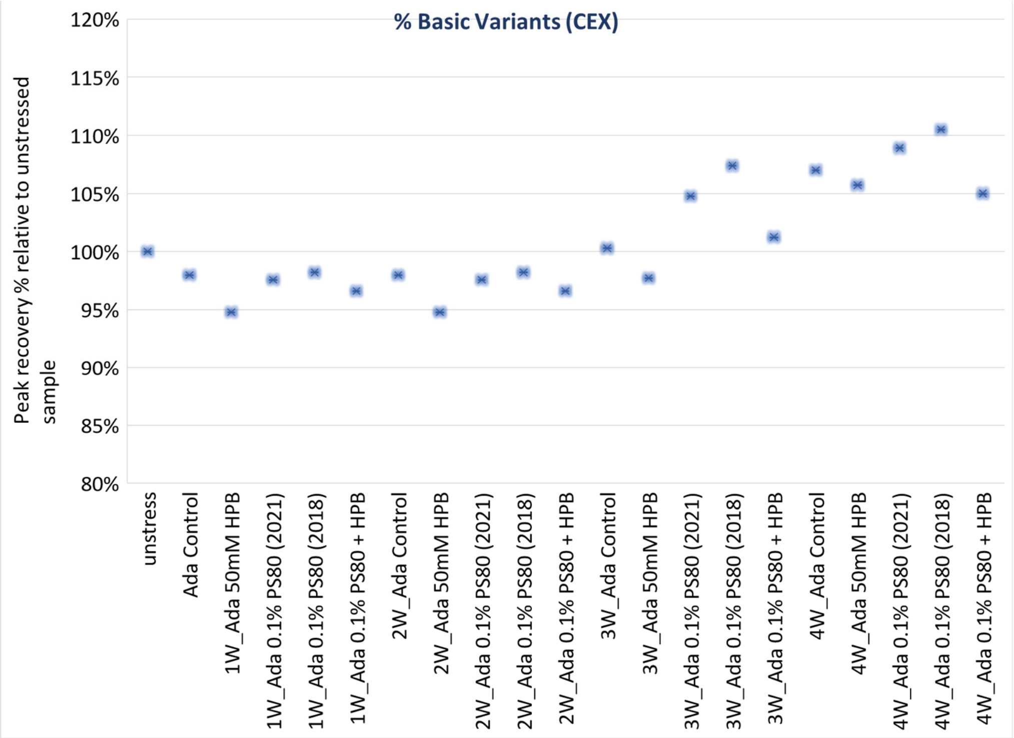

Supplementary Figure S4.2 Basic Species of adalimumab formulations under thermal and light stress conditions

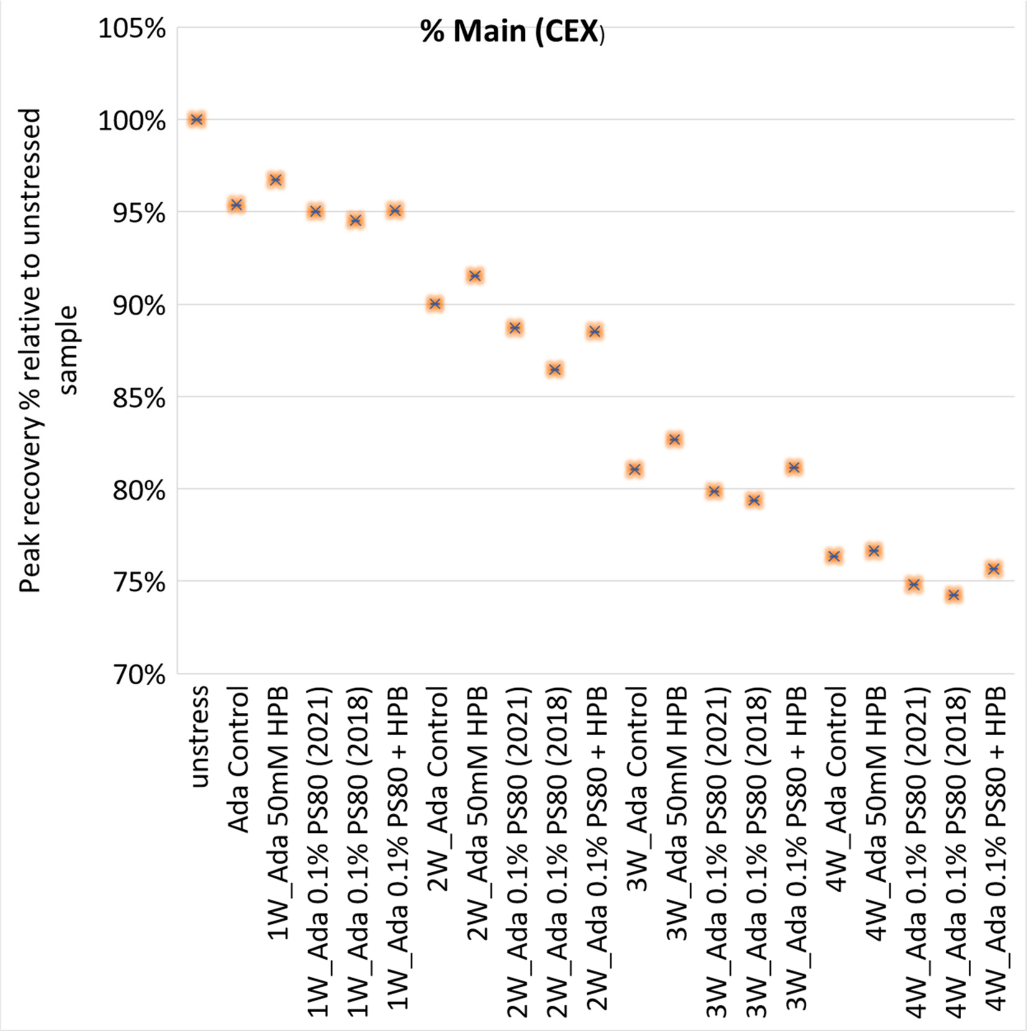

Supplementary Figure S4.3 Main Species of adalimumab formulations under thermal and light stress conditions
